# *C9ORF72* repeat expansion causes vulnerability of motor neurons to Ca^2+^-permeable AMPA receptor-mediated excitotoxicity

**DOI:** 10.1038/s41467-017-02729-0

**Published:** 2018-01-24

**Authors:** Bhuvaneish T. Selvaraj, Matthew R. Livesey, Chen Zhao, Jenna M. Gregory, Owain T. James, Elaine M. Cleary, Amit K. Chouhan, Angus B. Gane, Emma M. Perkins, Owen Dando, Simon G. Lillico, Youn-Bok Lee, Agnes L. Nishimura, Urjana Poreci, Sai Thankamony, Meryll Pray, Navneet A. Vasistha, Dario Magnani, Shyamanga Borooah, Karen Burr, David Story, Alexander McCampbell, Christopher E. Shaw, Peter C. Kind, Timothy J. Aitman, C. Bruce A. Whitelaw, Ian Wilmut, Colin Smith, Gareth B. Miles, Giles E. Hardingham, David J. A. Wyllie, Siddharthan Chandran

**Affiliations:** 1 MRC Centre for Regenerative Medicine, University of Edinburgh, Edinburgh, EH16 4UU UK; 2 Euan MacDonald Centre for MND Research, University of Edinburgh, Edinburgh, EH16 4SB UK; 3 Centre for Clinical Brain Sciences, University of Edinburgh, Edinburgh, EH16 4SB UK; 4 Centre for Discovery Brain Sciences, University of Edinburgh, Edinburgh, EH8 9XD UK; 5 School of Psychology and Neuroscience, University of St Andrews, St Andrews, KY16 9JP UK; 6 Centre for Brain Development and Repair, inStem, Bangalore, 560065 India; 7 The Roslin Institute and R(D)SVS, University of Edinburgh, Edinburgh, EH25 9RG UK; 8 Maurice Wohl Clinical Neuroscience Institute, King’s College London, London, SE5 8AF UK; 9 Global Biomarker and Drug Discovery, Biogen, Cambridge, MA 02142 USA; 10 Neurology Research, Biogen, Cambridge, MA 02142 USA; 11 MRC Institute of Genetics and Molecular Medicine, University of Edinburgh, Edinburgh, EH4 2XU UK; 12 UK DRI Institute at Edinburgh, University of Edinburgh, Edinburgh, EH16 4UU UK

## Abstract

Mutations in *C9ORF72* are the most common cause of familial amyotrophic lateral sclerosis (ALS). Here, through a combination of RNA-Seq and electrophysiological studies on induced pluripotent stem cell (iPSC)-derived motor neurons (MNs), we show that increased expression of GluA1 AMPA receptor (AMPAR) subunit occurs in MNs with *C9ORF72* mutations that leads to increased Ca^2+^-permeable AMPAR expression and results in enhanced selective MN vulnerability to excitotoxicity. These deficits are not found in iPSC-derived cortical neurons and are abolished by CRISPR/Cas9-mediated correction of the *C9ORF72* repeat expansion in MNs. We also demonstrate that MN-specific dysregulation of AMPAR expression is also present in *C9ORF72* patient post-mortem material. We therefore present multiple lines of evidence for the specific upregulation of GluA1 subunits in human mutant *C9ORF72* MNs that could lead to a potential pathogenic excitotoxic mechanism in ALS.

## Abstract (web summary)

Repeat expansion mutation in *C9ORF72* is the most common cause of familial ALS. Here, the authors generate motor neurons from cells of patients with *C9ORF72* mutations, and characterize changes in gene expression in these motor neurons compared to genetically corrected lines, which suggest that glutamate receptor subunit GluA1 is dysregulated in this form of ALS.

## Introduction

ALS is a relentlessly progressive and invariably fatal neurodegenerative disorder characterized by the selective death of MNs. Recognition that familial and sporadic ALS are largely phenotypically indistinguishable and share pivotal pathological features highlights the value of the study of familial ALS to shed mechanistic insight into ALS. Expansion of a GGGGCC (G_4_C_2_) intronic hexanucleotide in the *C9ORF72* gene is the most common cause of familial ALS and accounts for ∼10% of sporadic ALS cases^1,2^.

Although the precise underlying mechanisms of cell-type specificity and death is unknown in ALS, enhanced MN vulnerability to glutamate-mediated excitotoxicity leading to intracellular Ca^2+^ overload has been implicated^3–7^. Rodent studies have suggested that stoichiometry of AMPAR subunits is central to excitotoxicity noting that GluA1, 3, and 4 AMPAR subunits are Ca^2+^-permeable and that GluA2 subunit typically is Ca^2+^-impermeable^8–14^. Inefficient RNA editing of the GluA2 subunit, resulting in Ca^2+^-permeability, in sporadic ALS patient samples further highlights the importance of improved understanding of the role of AMPARs in ALS^15,16^.

A powerful approach to interrogate the consequences of G_4_C_2_ expansion in human MNs is the study of iPSC-derived MNs from ALS patients carrying the *C9ORF72* mutation^17,18^. Generation of isogenic lines using gene editing technologies, where the only variable is the mutation of interest, thus allowing direct causality to be assigned to any phenotype, further extends the biological relevance of iPSC studies^19–22^. In this study, we have used CRISPR/Cas9 technology to excise G_4_C_2_ repeats from ALS patient-derived MNs to enable direct study of the pathogenic role of G_4_C_2_ repeats with particular reference to AMPAR-mediated excitoxicity. We show that mutant *C9ORF72* MNs exhibit increased GluA1 AMPAR expression leading to enhanced MN-specific vulnerability to excitoxicity. This vulnerability is abolished by CRISPR/Cas9-mediated correction of the *C9ORF72* repeat expansion in MNs. Finally, we demonstrate that AMPAR expression is also selectively dysregulated in spinal cord, but not cortical, post-mortem tissue from patients harboring *C9ORF72* repeat expansions.

## Results

### Gene targeting and correction of the *C9ORF72* mutation

**Fig. 1:**
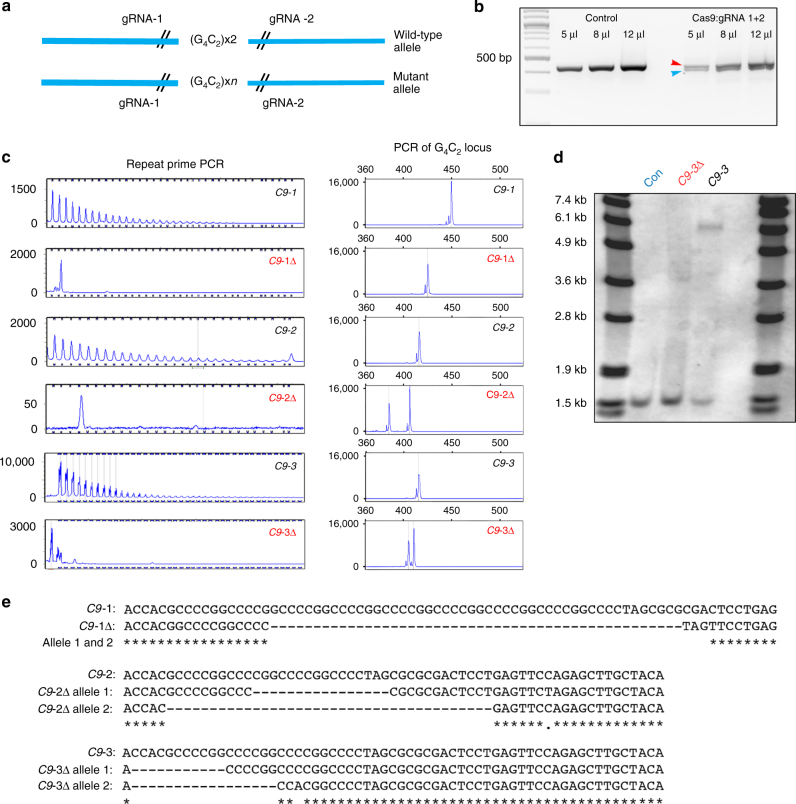
Excision of the G_4_C_2_ repeat expansion. **a** Strategy for generating C9-Δ from iPSC harboring G_4_C_2_ repeat expansion mutation. Two gRNAs were designed flanking the G_4_C_2_ repeats in intron-1 of the *C9ORF72* gene to generate a double-strand break (depicted by //). **b** PCR of the G_4_C_2_ locus in HEK-293 cells transfected with Cas9-gRNA-1 and 2 plasmids generated truncated PCR product (blue arrow) depicting both gRNAs are functional. **c** Repeat primed PCR and PCR flanking G_4_C_2_ locus showed absence of hexanucleotide repeat expansion in C9-Δ iPSC. **d** Southern blot analysis of *C9ORF72* alleles in control, mutant *C9ORF72*, and C9-Δ iPSC lines. **e** Sanger sequencing of G_4_C_2_ locus in C9ORF72 gene-edited lines confirmed excision of hexanucleotide repeat expansion in mutant allele. G_4_C_2_ repeats in wild-type allele were also excised by CRISPR/Cas9 editing strategy (C9-1: 6 repeats, C9-2: 2 repeats, C9-3: 1 repeat)

A CRISPR/Cas9-mediated targeting strategy was used to correct the mutation in three independent *C9ORF72* iPSC lines. Two CRISPR guide (g) RNAs (gRNA-1 and gRNA-2) were engineered to target either side of the G_4_C_2_ repeats in the intron1 of the *C9ORF72* gene. We selected gRNAs that have at least three mismatches to potential off-target sites, which are highly homologous to on-target sites. In the absence of a repair template the double-strand breaks were religated by non-homologous end joining resulting in deletion of G_4_C_2_ repeats (Fig. 1a). First, gRNAs (1 and 2) along with Cas9 plasmid were transfected in HEK-293 cells, which have 3 × G_4_C_2_ repeats, to test if the gRNAs were functional. PCR amplification of G_4_C_2_ locus in control HEK-293 cells generated a 420 bp amplicon, whereas HEK-293 cells transfected with gRNAs and Cas9 generated both an unedited 420 bp amplicon (red arrowhead) and a truncated PCR amplicon (blue arrowhead) suggesting both gRNAs were able to target and delete G_4_C_2_ repeats (Fig. 1b). Given that the gRNAs were functional, we next nucleofected Cas9 and gRNAs (1 and 2) into the mutant *C9ORF72* iPSC lines that harbor G_4_C_2_ repeats (C9-1, up to ~638 repeats; C9-2, up to ~960 repeats; C9-3, up to ~760 repeats; Supplementary Fig. 1A). Repeat primed PCR and G_4_C_2_ locus PCR were used to screen individual single-cell-derived clones. PCR of G_4_C_2_ locus in mutant *C9ORF72* iPSCs lines yielded a single amplicon of ~420–450 bp from the wild-type allele (C9-1, 8 repeats; C9-2, 2 repeats; C9-3, 2 repeats). The mutant allele is not amplified due to larger amplicon size. Deletion of the repeat expansion by CRISPR/Cas9 yields additional PCR amplicons (Fig. 1c). Efficiency of deletion of G_4_C_2_ repeats varied between the different iPSC lines ranging from 0.6–4.5% (C9-1, 2.27%; C9-2, 4.5%; C9-3, 0.6%). For each mutant *C9ORF72* iPSC line, we selected 1 clone that was confirmed by repeat primed PCR to have a lack of G_4_C_2_ repeats. All such lines are hereafter termed C9-1Δ, C9-2Δ, and C9-3Δ (Fig. 1c). In all three *C9ORF72* repeat expansion edited lines the wild-type allele was also modified since we observed truncated PCR amplicons (Fig. 1c). Southern blot analysis using a probe outside the G_4_C_2_ repeats confirmed absence of repeat expansion in the positive clone (Fig. 1d). We further sequenced the G_4_C_2_ locus of both the alleles in the positive clones to determine the deleted sequence. C9-1Δ contains 2 × G_4_C_2_ repeats in both alleles; C9-2Δ contains 1 × G_4_C_2_ repeat in one allele and absence of G_4_C_2_ repeat in other allele; and C9-3Δ contains 2 × G_4_C_2_ repeats in one allele and 1 G_4_C_2_ repeat in the other allele (Fig. 1e). This confirmed that the gene-corrected iPSC clone was karyotypically normal (Supplementary Fig. 1B) and retained pluripotency markers such as OCT3/4, TRA-1-60 (Supplementary Fig. 1C). RNA-guided genome editing, although efficient, has higher off-target activity when compared to other genome editing tools^23^. Assessment of highest candidate off-target sites, two for gRNA-1 and two for gRNA-2, by Sanger sequencing confirmed no off-target activity for the chosen gRNAs in all three mutant *C9ORF72* iPSC lines (Supplementary Fig. 1D).

### *C9ORF72* mutant MNs display RNA foci

**Fig. 2:**
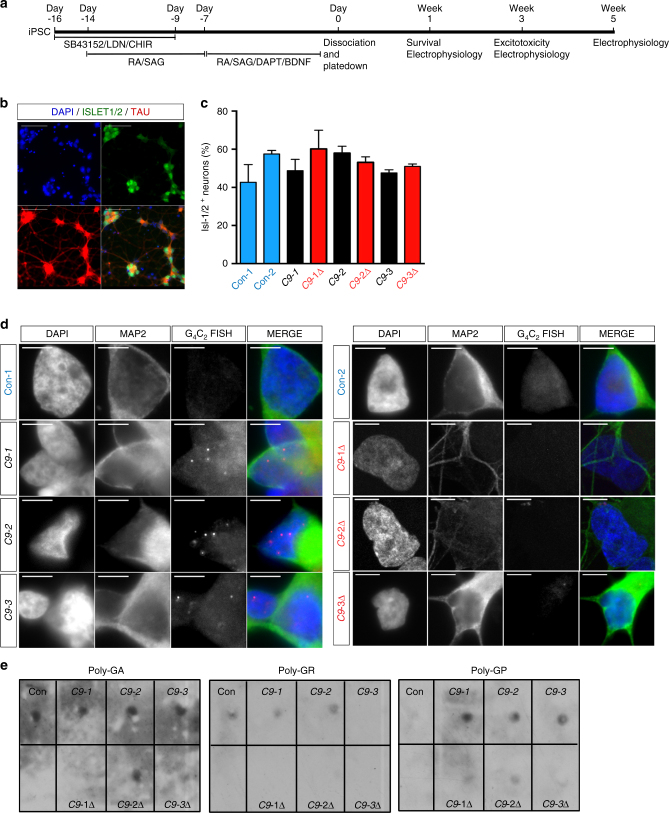
Excision of the G_4_C_2_ repeat expansion reverses RNA foci pathology in mutant MNs. **a** Schematic representation of MN generation and timings of various experiments performed. **b** Representative images of Isl1/2 and Tau immunostainings in 1-week-old MNs (scale bar: 50 µm). **c** Percentage of Isl1/2^+^ MNs from various iPSC lines (data represented as mean ± s.e.m., Con-1, *N* = 3; Con-2, *N* = 3; C9-1, *N* = 3; C9-1Δ, *N* = 3; C9-2, *N* = 3; C9-2Δ, *N* = 2; C9-3, *N* = 3; C9-3Δ, *N* = 3). **d** RNA fluorescent in situ hybridization showed mutant *C9ORF72* MNs exhibit intra-nuclear RNA foci (scale bar: 5 µm). **e** Representative dot-blots for poly-GA, poly-GP, and poly-GR DPRs in mutant *C9ORF72* MNs. RA retinoic acid, SAG smoothened agonist, DAPT N-[N-(3,5-Difluorophenacetyl)-L-alanyl]-S-phenylglycine t-butyl ester, BDNF Brain-derived neurotrophic factor

Having corrected the mutation, we next generated, using a recently reported protocol^24^, MNs from two unrelated healthy controls (Con-1, Con-2) and from three patients harboring the *C9ORF72* mutation (C9-1, C9-2, C9-3) and their isogenic pairs (C9-1Δ, C9-2Δ, C9-3Δ) (Fig. 2a). All lines efficiently differentiated into highly enriched neuronal cultures (>95% Tau^+^, devoid of astrocytes) that expressed the MN markers Isl1/2 (40–60%) and ChAT at 1 and 3 weeks post-plating, respectively (Fig. 2b, c; Supplementary Fig. 2A, B). Three principal hypotheses have been proposed to explain how mutant *C9ORF72* is pathogenic: haploinsufficiency leading to reduced levels of C9ORF72 protein, RNA foci binding essential RNA-binding proteins, and direct toxicity of dipeptide repeats (DPR) generated by repeat-associated non-ATG (RAN) translation of G_4_C_2_ repeats^25^. To evaluate whether expansion results in loss of protein, western blot analysis was undertaken. This revealed comparable amounts of C9ORF72 protein expression in MNs derived from all control, mutant, and gene-corrected iPSC lines (Supplementary Figs. 2C, 8B). Intranuclear RNA foci in MNs are a characteristic pathological finding in post-mortem tissue of *C9ORF72* patients^1^. Using fluorescent in situ hybridization we confirmed abundant intranuclear RNA foci in mutant MNs that were absent in controls and gene-corrected lines (Fig. 2d) and absent in RNAse-treated cultures. Recent reports have observed DPRs in mutant C9ORF72 material and indeed protein lysates from mutant *C9ORF72* MNs expressed higher levels of GA, GP, and GR RAN peptides when compared to control and gene-corrected MNs (Fig. 2e). Collectively, these findings demonstrate a direct causal link between *C9ORF72* mutation and the presence of intranuclear foci and RAN peptides. Next, we undertook survival analysis to determine if the *C9ORF72* mutation confers selective cellular vulnerability on MNs under basal conditions. Survival, measured using either single-cell longitudinal analysis or absolute cell counts, showed no difference between control and mutant MNs (Supplementary Fig. 2D). Thus, although mutant MNs display pathological RNA foci and DPRs these do not impact upon cell viability or survival under basal conditions.

### Mutant *C9ORF72* MNs display no difference in excitability

**Fig. 3:**
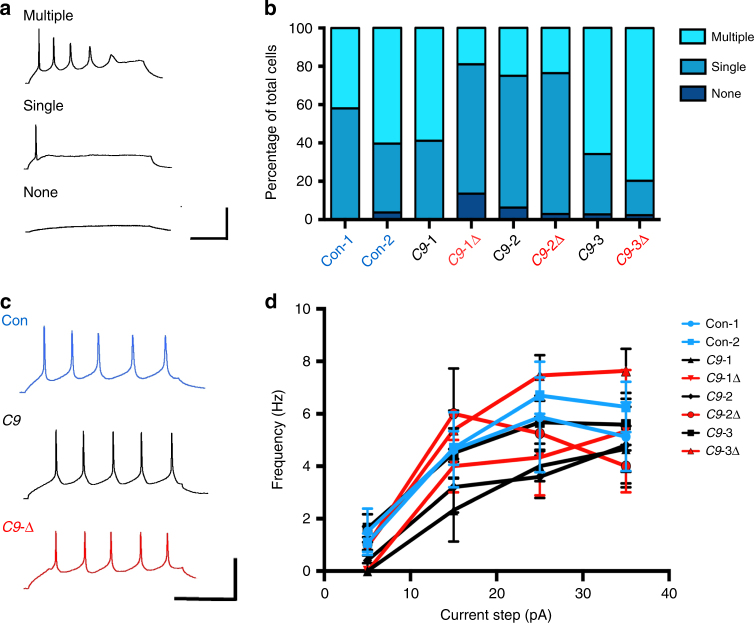
Excitability of MNs derived from mutant *C9ORF72* patient and control iPSCs. **a** Evoked firing types observed in iPSC-derived MNs. MNs displayed either multiple, single, or no action potentials upon current stimulation. **b** Percentage of cells showing each category of firing did not differ between MNs derived from control (Con-1, *n* = 31; Con-2, *n* = 53), *C9ORF72* mutant (C9-1, *n* = 17; C9-2, *n* = 32; C9-3, *n* = 73), and gene-edited (C9-1∆, *n* = 37; C9-2∆,*n* = 34; C9-3∆, *n* = 84) iPSC lines. **c** Raw traces showing repetitive action potential firing (>5 action potentials) in response to current injection in MNs derived from control, *C9ORF72* mutant, and gene-edited iPSC lines. **d** F–I relationships generated from repetitively firing MNs reveal no significant differences between MNs derived from control (Con-1, *n* = 8; Con-2, *n* = 23), *C9ORF72* mutant (C9-1, *n* = 3; C9-2, *n* = 5; C9-3, *n* = 22), and gene-edited (C9-1∆, *n* = 3; C9-2∆, *n* = 4; C9-3∆, *n* = 39) iPSC lines (mean ± s.e.m. values are plotted in the graph). Calibration bars: **a**: 200 ms, 20 mV; **c**: 400 ms, 20 mV

We and others have previously shown that mutant *C9ORF72* iPSC-derived MNs exhibit differences in excitability when using differentiation protocols which result in mixed cultures of neurons and glia^17,26,27^. In the present study using distinct protocols that produce neuronal only, MN-enriched cultures, whole-cell patch-clamp recordings of MNs were undertaken to assess their firing properties (Fig. 3a). No significant differences were observed in the proportions of firing types (Fig. 3b) or intrinsic properties (Supplementary Table 1) of MNs derived from control or mutant iPSCs. Frequency–current (F–I) relationships were also comparable between control and mutant *C9ORF72* MNs (Fig. 3c, d; Supplementary Table 1). Collectively, these findings reveal no difference in excitability between control and mutant *C9ORF72* MNs in MN-enriched cultures lacking glial cells and therefore implicate aberrant neuron–glia interactions in the functional deficits reported previously^17,26,27^.

### Altered GluA1 subunit expression in mutant *C9ORF72* MNs

**Fig. 4:**
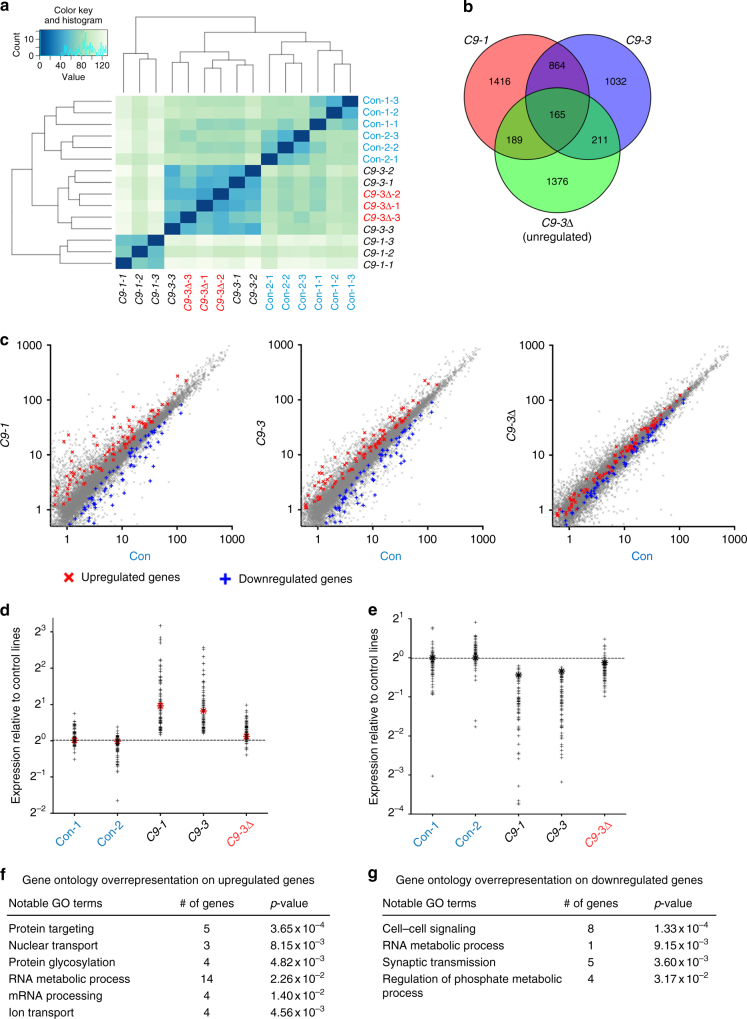
RNA-Seq analysis on *C9ORF72* mutant MNs. **a** Heat map and dendrogram depicting hierarchical clustering of RNA sequencing reads from controls (Con-1, Con-2), *C9ORF72* mutant (C9-1, C9-3), and C9-3Δ MNs. **b** Venn diagram depicting identification of differentially expressed genes between two mutant *C9ORF72* MNs and control MNs. Central intersect represents 165 dysregulated genes compared to both control and gene-corrected MNs. **c** Scatter plot showing comparison of transcriptome reads of two independent *C9ORF72* mutant MNs and C9-3Δ MNs to controls. Red crosses denote significantly upregulated genes and blue crosses denote significantly downregulated genes (*p* < 0.05, analysis performed using DESeq2). These dysregulated genes in *C9ORF72* mutant MNs are reversed to control levels in C9-Δ MNs. **d**, **e** Graph depicting the spread of differentially regulated, upregulated (**d**), and downregulated (**e**) genes in mutant *C9ORF72* MNs, red star denotes the levels of GluA1. **f**, **g** Gene ontology studies on upregulated (**f**) and downregulated genes (**g**)

To begin to understand the molecular consequences of *C9ORF72* mutation on MNs, we next undertook RNA-Seq analysis. Hierarchical clustering and heat map analysis showed segregation between control (including MN from an isogenic line, C9-3Δ) and mutants (C9-1, C9-3) (Fig. 4a). The high degree of similarity between C9-3 and C9-3Δ further indicates comparison between isogenic control overcomes the transcriptional heterogeneity observed in different iPSC lines and observed differential expression is driven by the mutation (Fig. 4a). We filtered differentially expressed genes using the following stringent criteria: (1) genes must have significant differential expression in both independent mutant *C9ORF72* lines (*p* < 0.05) when compared to independent control lines, (2) there must be significant difference between C9-3 and C9-3Δ (*p* < 0.05), and (3) no significant difference observed between C9-3Δ and controls (*p* > 0.05). Using this approach 85 upregulated and 80 downregulated genes, collectively 165, were identified (Fig. 4b, c, d; Supplementary Data 1, 2). Gene ontology analysis revealed that genes involved in RNA processing, protein targeting, nuclear transport, and ion transport were significantly upregulated in mutant *C9ORF72* MNs. Among downregulated genes in mutant *C9ORF72* MNs there was enrichment for genes involved in synaptic transmission, cell–cell signaling, and regulation of phosphate metabolism (Fig. 4f, g).

**Fig. 5:**
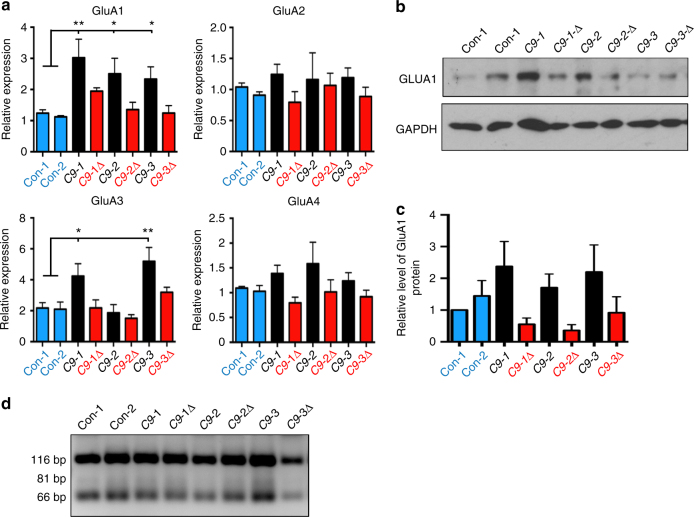
The *C9ORF72* mutation is associated with increased expression of GluA1 AMPAR subunits. **a** Relative mRNA expression of AMPAR subunits in all cultures examined at week 3 after differentiation (data represented as mean ± s.e.m., Con-1, *N* = 5; Con-2, *N* = 6; C9-1, *N* = 8; C9-1Δ, *N* = 4; C9-2, *N* = 7; C9-2Δ, *N* = 6; C9-3, *N* = 7; C9-3Δ, *N* = 6). Statistical comparisons; one-way ANOVA with uncorrected Fisher’s LSD. Significant *p*-values for GluA1 C9-1 vs. Con-1: 0.0039, C9-2 vs. Con-1: 0.0360, C9-3 vs. Con-1: 0.0503, C9-1 vs. Con-2: 0.0130, C9-2 vs. Con-2: 0.0163, C9-3 vs. Con-2: 0.0385. *p*-values for GluA3 C9-1 vs. Con-1: 0.0218, C9-3 vs. Con-1: 0.0017, C9-1 vs. Con-2: 0.0256, C9-3 vs. Con-2: 0.0024. **b** Representative immunoblot showing elevated levels of GluA1 in *C9ORF72* mutant MNs. **c** Quantification of GluA1 protein level in *C9ORF72* mutant MNs (data represented as mean ± s.e.m., Con-1, *N* = 3; Con-2, *N* = 5; C9-1, *N* = 6; C9-1Δ, *N* = 3; C9-2, *N* = 3; C9-2Δ, *N* = 3; C9-3, *N* = 6; C9-3Δ, *N* = 6). **d** Representative gel picture showing efficient GluA2 Q→R RNA editing in week 3 cultures across all the lines. Efficient GluA2 RNA editing results in RFLP amplicons of 116 and 66 bp, see also Supplementary Fig. 4B. Band at 81 bp would be observed if inefficient editing of the GluA2 subunit was present

The finding that the GluA1 subunit was significantly upregulated (>2-fold) in *C9ORF72* MNs (red star, Fig. 4d) is consistent with multiple lines of evidence implicating dysregulation of glutamate receptor homeostasis in the pathogenesis of ALS^28^. RNA-Seq analysis of the other AMPAR subunits and their auxiliary subunits showed that only GluA1 mRNA was differentially expressed in mutant *C9ORF72* MNs (Supplementary Fig. 3). Subsequent RT-qPCR confirmed increased expression of GluA1 in all week 3 mutant *C9ORF72* MNs with gene-corrected lines showing reversal to control levels. Moreover, we also observed increases in GluA3 mRNA expression in mutant *C9ORF72* MNs for two out of the three lines examined (Fig. 5a). Western blot analysis also confirmed increased levels of GluA1 in all week 3 mutant *C9ORF72* MNs with gene-corrected lines displaying levels comparable to controls (Fig. 5b, c; Supplementary Fig. 8A). Importantly, relative expression of GluA2 mRNA was not altered in any of the lines (Fig. 5a). As would be expected, given the neuronal maturation profile of AMPAR subunit composition^29^, we did not observe altered expression of Ca^2+^-permeable AMPAR subunits in week 1 MNs (Supplementary Fig. 4A).

The increased proportion of Ca^2+^-permeable AMPARs in mutant MNs at week 3 could also be due to inefficient RNA editing of GluA2 subunit in addition to increased expression of non-GluA2 subunits. We found, however, that GluA2 subunits were efficiently RNA-edited in all mutant cultures (Fig. 5d; Supplementary Fig. 4B). Together, these findings demonstrate that mutant *C9ORF72* MNs exhibit an increased expression of Ca^2+^-permeable GluA1 AMPAR subunits, but not inefficient editing of the GluA2 subunit.

### Mutant *C9ORF72* MNs are more vulnerable to excitotoxicity

**Fig. 6:**
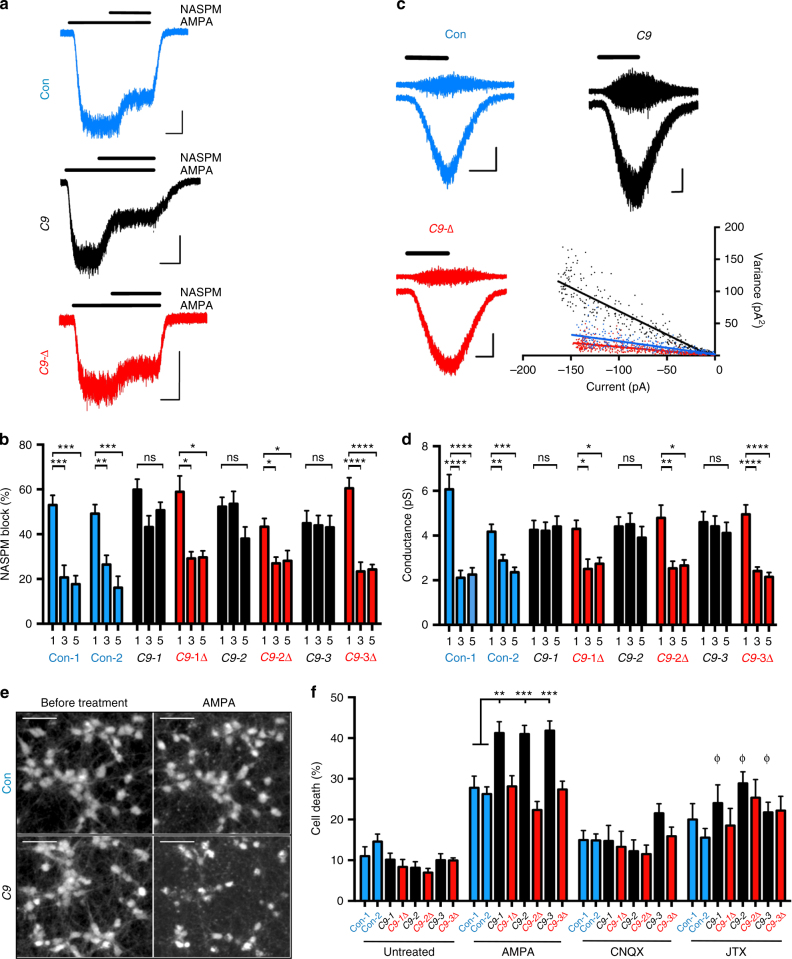
Mutant *C9ORF72* MNs exhibit Ca^2+^-permeable AMPARs receptors and greater vulnerability to excitotoxicity. **a** Sample whole-cell voltage-clamp recordings conducted at −74 mV depicting the block of AMPA (10 μM)-mediated currents (in the presence of cyclothiazide (10 μM)) by Ca^2+^-permeable AMPAR-selective blocker NASPM (3 μM) in week 5 MNs derived from a control, mutant *C9ORF72*, or C9*-*Δ lines. Scale bars: 5 s, 50 pA. **b** Mean ( ± s.e.m.) percentage NASPM block of AMPA currents in all MNs examined at week 1, 3, and 5 (data for week 1/week 3/week 5; Con-1, *n* = 6/10/6, *N* = 3/3/1; Con-2, *n* = 14/12/6, *N* = 2/2/2; C9-1, *n* = 9/9/14, *N* = 2/1/3; C9-1Δ, *n* = 5/8/5, *N* = 2/2/2; C9-2, *n* = 9/12/12, *N* = 2/3/3; C9-2Δ, *n* = 8/10/10, *N* = 3/4/4; C9-3, *n* = 7/11/11, *N* = 3/3/3; C9-3Δ, *n* = 10/10/7, *N* = 3/3/3) after differentiation (statistical comparisons; one-way ANOVA with Bonferroni’s post hoc test). **c** Representative non-stationary fluctuation analysis recordings of AMPAR-mediated currents in the AC-current (upper) and DC-current modes (lower) of week 5 MNs derived from a control, mutant *C9ORF72*, or the C9*-*Δ lines. Current-variance plots (bottom right) generated from the recordings presented were used to derive the *γ*. Scale bars; 5 s, 40 pA. **d** Mean estimated AMPAR γ in all lines examined at week 1, 3, and 5 (data week 1/week 3/week 5; Con-1, *n* = 15/15/12, *N* = 4/4/3; Con-2, *n* = 13/15/8, *N* = 2/2/2; C9-1, *n* = 10/11/21, *N* = 2/3/3; C9-1Δ, *n* = 7/6/8, *N* = 2/2/2; C9-2, *n* = 15/15/13, *N* = 3/3/2; C9-2Δ, *n* = 11/12/7, *N* = 3/3/2; C9-3, *n* = 12/18/19, *N* = 4/3/4; C9-3Δ, *n* = 17/23/8, *N* = 4/4/2) after differentiation (statistical comparisons; one-way ANOVA with Bonferroni’s post hoc test). **e** Representative images of Calcein AM-stained neurons derived from control, *C9*, and C9*-*Δ lines before and after 24-h treatment of AMPA (100 μM) in the presence of cyclothiazide (100 µM). Scale bar: 50 µm. Cyclothiazide is an AMPAR-selective allosteric potentiator used to prevent rapid AMPAR desensitization. **f** Mean percentage cytotoxicity of MNs treated with AMPA, AMPA + JTX, and AMPA + CNQX (data represented as mean ± s.e.m., Con-1, *N* = 6; Con-2, *N *= 5; C9-1, *N* = 5; C9-1Δ, *N* = 5; C9-2, *N* = 9; C9-2Δ, *N* = 5; C9-3, *N* = 5; C9-3Δ, *N* = 5) and at least 100 cells per experiment, Greek letter phi denotes significant difference (two-way ANOVA with uncorrected Fisher's LSD) in cell death with JTX treatment  when compared to AMPA treatment

To determine whether the observed changes in the AMPAR subunit expression profiles had physiological consequences we next examined whether week 3 mutant MNs exhibited enhanced relative functional expression of Ca^2+^-permeable AMPARs. MNs from all lines expressed robust AMPA-evoked currents that were potentiated by the allosteric modulator, cyclothiazide, and blocked by AMPAR antagonist CNQX (Supplementary Fig. 5A). The presence of one or more edited GluA2(R) subunits in the AMPAR complex confers Ca^2+^-impermeability, reduced single-channel conductance, and reduced sensitivity to channel-blocking polyamines^29,30^. We assessed the proportion of Ca^2+^-permeable AMPARs in MNs by measuring the block of AMPAR-mediated currents by NASPM, a selective blocker of Ca^2+^-permeable AMPARs (Fig. 6a, b), and estimated their mean single-channel conductance using non-stationary fluctuation analysis (Fig. 6c, d). Comparison of weeks 1 and 3 control MNs showed a reduction in both mean NASPM block and single-channel conductance that is consistent with a transition to a predominantly Ca^2+^-impermeable AMPAR population (Fig. 6b, d) and the maturation of AMPAR composition in native MNs^29^. In contrast, mutant *C9ORF72* MNs at week 3 retained both a high sensitivity to NASPM block and an elevated single-channel conductance consistent with persistent expression of Ca^2+^-permeable AMPARs (Fig. 6b, d). Strikingly, the maturation of AMPAR composition in C9-1Δ, C9-2Δ, and C9-3Δ MNs reflected that of control MNs. Importantly the retention of Ca^2+^-permeable AMPARs in mutant *C9ORF72* MNs was not due to a slower maturation of AMPAR composition as prolonging culture times for a further 2 weeks did not reveal any changes in the biophysical properties of AMPARs (Fig. 6b, d). Furthermore, we did not observe any impairment in the maturation of intrinsic properties or excitability of MNs across all lines, indicating that AMPAR-mediated-induced firing is unlikely to be a differential source of excitotoxicity in our cultures. These data demonstrate that the *C9ORF72* mutation increases persistence in the relative expression of Ca^2+^-permeable AMPARs.

Given the alteration in AMPAR composition we next examined whether *C9ORF72* patient-derived MNs showed increased vulnerability to AMPA-induced excitotoxicity. Exposure to AMPA resulted in greater levels of cell death in week 3, but not week 1, mutant *C9ORF72* MN cultures vs. control and C9*-*Δ cultures (Fig. 6e, f; Supplementary Fig. 5B, C). Importantly, enhanced susceptibility of mutant *C9ORF72* MNs to excitotoxicity was not due to elevated AMPAR current flux as evidenced by no differences in AMPA-mediated current densities in each of the cell lines at week 3 (Supplementary Fig. 5D). Excitotoxicity experiments performed in the presence of joro toxin (JTX), a selective blocker of Ca^2+^-permeable AMPARs^31^ significantly reduced cell death of week 3 mutant MNs (Fig. 6f). These data therefore indicate the *C9ORF72* mutation is associated with a higher susceptibility to excitotoxicity due an increase in relative expression of Ca^2+^-permeable AMPARs through altered AMPAR subunit expression.

To determine whether persistent expression of Ca^2+^-permeable AMPAR was selective to MNs we next generated cortical neurons from Con-1, C9-3, and C9-3Δ iPSC lines (Supplementary Fig. 6A, B, C)^32,33^. The extent of NASPM block of AMPAR-mediated currents and the estimated single-channel conductance of AMPARs in C9-3 cortical neurons, unlike MNs, did not demonstrate an elevated expression of Ca^2+^-permeable AMPARs (Supplementary Fig. 6D, E). Taken together these findings are consistent with a MN-specific cellular pathophysiology caused by the presence of mutant *C9ORF72*.

### Elevated GluA1 expression in spinal cord of ALS patients

**Fig. 7:**
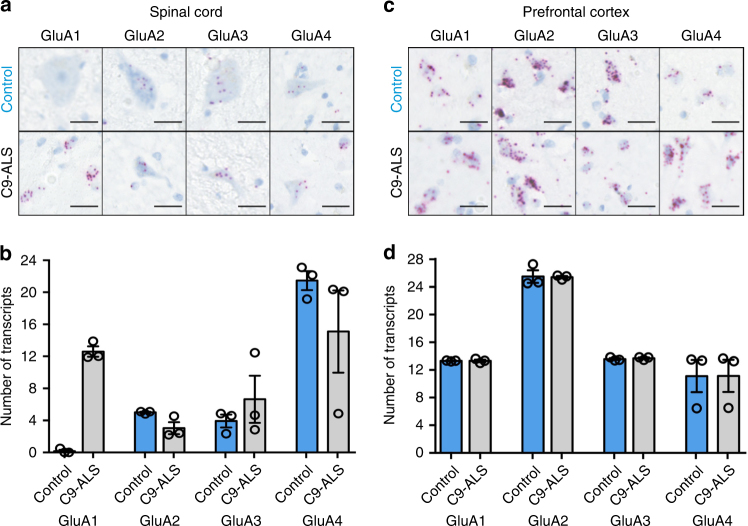
AMPAR subunit expression in post-mortem spinal cord of healthy patients and ALS patients with *C9ORF72* repeat expansion. **a** Representative RNAscope® images showing the expression of AMPAR GluA1—four transcripts in the anterior horn of cervical spinal cord sections from an ALS patient (*C9ORF72*) and an age-matched healthy patient and reveals increased expression of GluA1 in ALS patient sample. Scale bar, 25 µm. **b** Quantification of the mean ± s.e.m. expression levels of AMPAR transcripts in spinal cord samples from three independent patients and controls. Note the lack of GluA1 transcript expression in control spinal cord samples and strong expression in patient samples. **c** Representative RNAscope images showing the expression of AMPAR GluA1—four transcripts in the prefrontal cortex from an ALS patient (*C9ORF72*) and an age-matched healthy patient. Scale bar, 25 µm. **d** Quantification of the mean ± s.e.m. expression levels of AMPAR transcripts in prefrontal cortex samples from three independent patients and controls showing equivalent expression of all transcripts

To evaluate whether findings in iPSC-derived neurons are recapitulated in pathological samples from ALS patients, AMPAR subunit expression was next examined by RNAscope®. Post-mortem cervical spinal cord and prefrontal cortex (BA9) sections from three *C9ORF72* mutation-carrying ALS patients and three age-matched controls with no known neurodegenerative disease were treated with fluorescence in situ hybridization (FISH) probes against AMPAR subunits and the positive control enzyme PPIB (Supplementary Fig. 7). In agreement with previous reports of control samples displayed negligible GluA1 expression in adult MNs (Fig. 7a)^34,35^. However, high expression of GluA1 RNA was observed in the anterior horn neurons of all ALS patients (Fig. 7b). No difference was found in GluA2, 3, and 4 RNA expression between ALS and control spinal cord samples (Fig. 7c). Conversely, in prefrontal cortex sections, expression of all AMPAR RNA transcripts was similar between ALS and control samples (Fig. 7c, d). These data confirm in human post-mortem tissue that the *C9ORF72* expansion is associated with an increase in GluA1 RNA expression in spinal cord MNs but not in prefrontal cortical neurons.

## Discussion

The ability to study human disease-causing mutations under genetically defined conditions allows causal rather than correlative associations to be ascribed. Using CRISPR technology to correct the *C9ORF72* mutation from three patient-derived iPSC lines we show that the *C9ORF72* mutation is associated with an increase in GluA1 AMPAR subunit expression, functional expression of Ca^2+^-permeable AMPARs, and MN vulnerability to excitotoxicity. *C9ORF72* patient post-mortem tissue also demonstrates a marked selective upregulation in GluA1 RNA expression in spinal cord MNs. Both iPSC and pathological data further reveal that dysregulation of AMPAR expression is not found in cortical neurons and thus is MN specific.

There are three major hypotheses as to how *C9ORF72* repeat expansion might be pathogenic: loss of, as yet still unknown, normal C9ORF72 function, sequestration of RNA binding proteins by RNA foci, and direct toxicity mediated by DPRs^25^. These processes are not mutually exclusive. We observed RNA foci and DPRs in mutant MNs whose expression was reduced or abolished by gene correction^17,18,36^. These findings, consistent with the idea that RNA-binding proteins sequestered by RNA foci in mutant *C9ORF72* MNs dysregulate RNA metabolism, are supported by the differential transcriptional findings between mutant, control, and corrected MNs revealing an enrichment for genes involved in RNA metabolism, stress response, and nuclear transport. Collectively our data are consistent with recent studies including those from human pathological findings^17,18,37–39^. Moreover, our RNA-Seq data reveal new candidate genes that will require future validation and functional assessment. These include genes involved in synaptic transmission, protein targeting, and cell–cell signaling. In view of recent findings, it would be of interest to determine whether the increased expression of GluA1 is related to pathological RNA foci and/or DPRs^40^. Furthermore, it will be of considerable interest to study whether other genetic cause of ALS results in AMPAR dysregulation.

In mixed cultures of neurons and glia derived from *C9ORF72* repeat expansion patient iPSCs, MNs first exhibit intrinsic hyperexcitability^27^, then progress to a hypoexcitable state^17^. In contrast, here we have used a modified protocol for the generation of highly enriched neuronal cultures that contains negligible numbers of glial cells^24,41^. Under these conditions we found no difference in MN intrinsic excitability. A likely explanation for the lack of changes in excitability in the present study is that glial-mediated non-cell autonomous mechanisms underlie changes in MN function. This hypothesis is supported by recent findings in murine cell culture models where exposure of wild-type MNs to conditioned medium derived from astrocytes expressing mutant SOD1 alters ion channel function and MN excitability^42^.

Multiple lines of evidence suggest that dysregulated glutamate homeostasis plays a role in ALS. These include patient studies showing reduced expression of glial glutamate transporter, enhanced CSF glutamate levels, and in vitro and in vivo studies that reveal selective MN vulnerability to glutamate or AMPAR-mediated, but not NMDAR, excitotoxicity^3,4,43^. AMPAR subunit composition is dynamic and changes in development and is dysregulated in disease. In early post-natal life, there is a switch from predominantly Ca^2+^-permeable to Ca^2+^-impermeable AMPARs which reflects increased GluA2 containing AMPARs; the dominant functional determinant of AMPAR-mediated Ca^2+^-permeability^29^. Indeed, this maturation is recapitulated in this study with control and gene-corrected iPSC MNs showing a shift from predominant Ca^2+^-permeable to Ca^2+^-impermeable AMPAR from 1 to 3 weeks in culture. In contrast, mutant *C9ORF72* MNs display persistent high levels of Ca^2+^-permeability at 3 and 5 weeks in culture. The blockade of AMPA-mediated MN cell death by JTX treatment, a selective blocker of Ca^2+^-permeable AMPARs, confirms that *C9ORF72* mutation susceptibility to excitotoxicity is due to an increase in relative expression of Ca^2+^-permeable AMPARs. Our findings are consistent with an earlier study that showed an enhanced vulnerability of iPSC-derived mutant *C9ORF72* MNs to glutamate-mediated excitotoxicity^18^.

One of the proposed mechanisms of MN death in ALS is through inefficient post-transcriptional editing of the Q/R site within the GluA2 subunit with the resulting AMPAR becoming Ca^2+^-permeable^16,44^. Our finding of efficient RNA editing does not support reduced editing as an explanation for increased Ca^2+^-permeability in mutant *C9ORF72* MNs and is consistent with findings in native neurons and SOD1 familial cases^45,46^. Alternative explanations for increased Ca^2+^-permeability include disease-related alterations in AMPAR subunit composition. Experimental evidence in support of this idea is provided by findings in the SOD1 model of ALS, although these reports do not agree on the precise identity of dysregulated AMPAR subunits^8,10,11,47,48^. Against this background, we show multiple lines of evidence that demonstrate upregulation in the GluA1 AMPAR subunit in *C9ORF72* mutant MNs. The maintenance of functional expression of the GluA1 subunit strongly correlates with an increased Ca^2+^-permeable AMPAR identity in our cultures. Although GluA1 upregulation has previously been reported in the spinal cord and hippocampus of hSOD1G93A mice^11,49^, our study now demonstrates potential functional consequences of increased GluA1 in an experimental ALS model. Furthermore, our data demonstrate that mutant *C9ORF72* cortical neurons possess predominantly Ca^2+^-impermeable AMPARs, indicating that mutant *C9ORF72*-dependent increases in Ca^2+^-permeable AMPARs are selective to MNs. Moreover, our findings are consistent with recent reports from *C9ORF72* FTD patient iPSC neurons and post-mortem cortices that also do not show altered AMPAR subunit transcript regulation^37,50^. Furthermore, our earlier report that oligodendrocytes generated from the same mutant *C9ORF72* patient iPSC lines used in this study express predominantly Ca^2+^-impermeable AMPARs further highlights MN-specific consequence of the *C9ORF72* mutation^51^. Finally, in post-mortem studies, we show for the first time that mutant *C9ORF72* spinal cord but not cortical neurons show selective upregulation of GluA1 transcript by quantitative RNAscope compared to age-matched control samples. We therefore propose that the mutant *C9ORF72*-dependent increase in Ca^2+^-permeable AMPAR-mediated excitotoxicity is MN specific.

Importantly, our in vitro and post-mortem data suggest that GluA1 subunit upregulation may be contributory to selective MN death in ALS. Notwithstanding our observation of selective upregulation of GluA1 in post-mortem mutant *C9ORF72* spinal cord MNs, we are unable to determine whether this is causal or a consequence of a separate disease pathology. It is also important to note that our study cannot exclude other AMPAR-related mechanisms that could potentially play a role in mutant *C9ORF72*-related MN pathology^18^. Furthermore, the degree to which other mechanisms may concurrently contribute to excitotoxicity via elevated glutamate levels at synapses, such as changes in glial glutamate transporter expression remains unknown^52,53^.

Our RNA-Seq study uncovered a mutant *C9ORF72*-associated increase in GRIA1 (GluA1) expression, which led to the hypothesis of increased Ca^2+^-permeable AMPA receptor currents and elevated vulnerability to AMPAR-mediated excitotoxicity. However, other genes whose expression are perturbed may also contribute to this phenotype. Our data also revealed a *C9ORF72* mutation-associated reduction in PICK1 expression. PICK1 regulates the surface trafficking of GluA2-containing AMPARs^54^, and so a reduction in its expression may modify the proportion of GluA2-lacking AMPARs at the surface. Further genes within this data set may contribute to, or reflect other aspects of mutant *C9ORF72*-associated cellular phenotypes. For example, we observe a *C9ORF72* mutation-associated increase in HSPB8, SERP1, and HSP90B1. The small heat shock protein HSPB8/HSP22 plays a role in protein quality control and is capable of promoting the clearance of aggregates of C9ORF72 dipeptides, as well as mutant SOD1 and TDP-43^55–57^. SERP1 (RAMP4) is induced by ER stress via a XBP-1-dependent process^58^ whose role is to reduce protein stress by stabilizing proteins to facilitate glycosylation and promote translocation of proteins into the ER^59–61^. HSP90B1 (GRP94, GP96) is another ER chaperone involved in the unfolded protein response (UPR), and a ATF6 target^62^. Collectively, the induction of these genes point to a degree of ER stress and activation of the UPR. Conversely, increased ER Ca^2+^ levels, elevated GRP78/BiP^63^, and vulnerability to tunicamycin-induced ER stress/UPR^64^ have been observed in *C9ORF72* mutant motor neurons. This may be triggered by the presence of C9ORF72 dipeptides and/or C9ORF72 hexanucleotide-induced disruption of nucleocytoplasmic transport^65^ and even be exacerbated by increased GluA2-lacking AMPARs, since excessive Ca^2+^ influx can influence ER stress^66^. Also of interest, GluA1 surface expression is known to be promoted by the UPR^67^, raising the possibility of positive feedback on excitotoxic signaling.

In summary, this study has combined patient iPSC lines and CRISPR/Cas9 technologies to demonstrate that the *C9ORF72* repeat expansion recapitulates key pathological features of mutant C9ORF72 ALS and, with *C9ORF72* ALS patient post-mortem material, suggests a possible mechanism for MN vulnerability to AMPAR-mediated excitotoxicity.

## Methods

### Cloning of CRISPR/Cas9 constructs

Cas9 and gRNA were cloned into an expression plasmid pSpCas9(BB)-2A-GFP (pX458) following a published protocol^68^. Web tool (http://crispr.mit.edu) was used to design CRISPR gRNA sequences and identify potential homologous off-target sites. Sequences of gRNAs are as follows: gRNA-1 5′-AACTCAGGAGTCGCGCGCTAGGG-3′, gRNA-2 5′-GGCCCGCCCCGACCACGCCCCGG-3′.

### Derivation of iPSCs

Dermal fibroblasts from patient and control individuals were obtained under full Ethical/Institutional Review Board approval at the University of Edinburgh. Fibroblasts were reprogrammed to iPSCs by either Sendai virus or retrovirus expressing *OCT4*, *SOX2*, *C-MYC*, and *KLF4*. iPSCs were maintained in Matrigel (BD Biosciences)-coated plastic dishes in E8 medium (Life Technologies) at 37 °C and 5% CO_2_.

### Gene editing of iPSCs

iPSC were dissociated into single-cell suspension using 1 × Accutase (Sigma). 8 × 10^5^ cells were nucleofected with 2 µg each of Cas9-gRNA1 and Cas9-gRNA2 plasmid using the Amaxa 4D nucleofector system (program CA137) following the manufacturer’s instructions. Transfected cells were plated onto Matrigel (BD)-coated dishes in the presence of E8 medium supplemented with ROCK inhibitor (10 µM). Upon confluence, cells were dissociated again into single cells and plated down at low density (2000 cells/10 cm dish) for clonal analysis. Individual clones were picked and screened for deletion of G_4_C_2_ repeats by G_4_C_2_ locus PCR and repeat primed PCR using an ABI 3130xl genetic analyzer.

### Repeat primed PCR

The *C9ORF72* repeat expansion mutation was confirmed in both iPSCs and MNs by repeat primed PCR using Qiagen multiplex PCR kit. PCR components are as follows: 1 × Multiplex Mastermix, 7% dimethylsulfoxide, 0.6 M Betaine, 7.6 µM FAM-labeled RP Fw (5′-CTGTAGCAAGCTCTGGAACTCAGGAGTCG-3′), 3.6 µM RP Rev: (5′-TACGCATCCCAGTTTGAGACGCCCCGGCCCCGGCCCCGGCCCC-3′), 11.6 µM Tail Rev (5′-TACGCATCCCAGTTTGAGACG-3′), 8 mM 7-deaza-2′-dGTP, and 200 ng DNA. Cycling conditions were performed as per the manufacturer’s recommendations with annealing temperature of 68 °C for 15 cycles, followed by 60 °C for a further 20 cycles. PCR products were separated on an ABI 3130xl analyzer (Life Technologies) and data were analyzed using GeneMarker software (Soft Genetics).

A G_4_C_2_ flanking PCR for screening targeted clones was also performed using Qiagen multiplex PCR kit. The PCR reaction was set up as per manufacturer’s guide lines using the following primers G_4_C_2_ Fw: 5′-CAAGGAGGGAAACAACCG-3′ G_4_C_2_ Rev: 6-FAM- 5′-GGAAAGCAAGGAAGAGGC-3′. PCR products were separated on an ABI 3130xl analyzer (Life Technologies) and data were analyzed using GeneMarker software (Soft Genetics).

### Southern blot

Southern blot analysis was used to analyze the C9ORF72 repeat expansion mutation as described^69^. DNA was isolated using Wizard® genomic DNA purification kit (Promega). 20 µg of DNA was digested overnight with *Xba* endonuclease, separated on a 0.8% agarose gel electrophoresis, gel was subsequently depurinated, denatured, and transferred overnight on to a positively charged nylon membrane (Roche). The membrane was UV cross-linked and hybridized overnight at 47 °C with a digoxigenin (DIG)-labeled PCR probe. PCR probe was synthesized from genomic DNA using PCR DIG probe synthesis kit (Roche) using following primers South Fw: 5′-CTTGCAGATCAAAAGGCACA-3′ and South Rev: 5′-TTGACGCACCTCTCTTTCCT-3′. Following hybridization, membrane was washed and labeled with anti-DIG antibody and developed using CDP-Star reagent as per manufacturer’s recommendation (Roche).

### MN differentiation

MN differentiation from iPSCs was performed using established protocol^24^ with minor modifications. iPSCs were dissociated into single cells using 1 × Accutase, 2 × 10^6^ iPSCs were neuralized as a suspension culture using SB-431542 (20 µM), LDN-193189 (0.1 µM), and CHIR-99021 (3 µM) in N2/B27 media (0.5 × Neurobasal, 0.5 × Advanced DMEM/F12, 1 × Antibiotic-Antimycotic, 1 × Glutamax, 100 µM beta-mercaptoethanaol, 1 × B27, 1 × N2, and 10 µM ascorbic acid). On day 2, neural spheres were simultaneously patterned to spinal cord identity by treating with retinoic acid (RA, 0.1 µM) and smoothened agonist (SAG, 500 nM) along with SB-431542, LDN-193189, and CHIR-99021 in N2/B27 media for additional 5 days. On day 7, spheres continued to be cultured in RA and SAG and, also, brain-derived neurotrophic factor (BDNF, 10 ng/ml) in N2/B27 media to generate MN progenitors. On day 9, MN progenitors were cultured in the previous media in addition to DAPT (10 µM) for additional 5–7 days. At day 14–16, MN spheres were dissociated using 0.05% Trypsin-EDTA and plated on to a poly-ornithine, laminin (5 µg/ml), fibronectin (10 µg/ml), Matrigel (1:20)-coated dishes. Dissociated MNs were cultured in NB media (1 × Neurobasal, 1 × Glutamax, 1 × non-essential amino acids, 100 µM beta-mercaptoethanol, 1 × B27, 1 × N2, RA (1 µM), ascorbic acid (2.5 µM), BDNF (10 ng/ml), glial-derived neurotrophic factor (10 ng/ml), ciliary neurotrophic factor (10 ng/ml), insulin-like growth factor (10 ng/ml)). Cells were treated with Uridine (1 µM)/5-fluoro-2′-deoxyuridine (U/FDU) on day 1 to remove residual proliferating cells. Media was changed every 2–3 days.

### Quantitative RT-PCR

qPCRs were performed following previously optimized protocol^32^. Briefly, RNA from week-1 and week-3-old MNs were isolated using RNeasy kit (Qiagen). cDNA was synthesized from 250 ng of total RNA using DyNAmo^TM^ cDNA synthesis kit (Thermo Scientific). Real-time quantitative PCR reactions were set up using DyNAmo ColorFlash SYBR Green qPCR kit (Thermo Scientific) and performed on CFX96 system (BioRad). Primers (forward and reverse) and annealing temperature used in this study are as follows: *GRIA1*: 5′-TGCTTTGTCGCAACTCACAGA-3′, 5′-GGCATAGACTCCTTTGGAGAAC-3′, 64 °C; *GRIA2*: 5′-CATTCAGATGAGACCCGACCT-3′, 5′-GGTATGCAAACTTGTCCCATTGA-3′, 58 °C; *GRIA3*: 5′-ACCATCAGCATAGGTGGACTT-3′, 5′-GGTTGGTGTTGTATAACTGCACG-3′, 58 °C; *GRIA4*: 5′-TTCCGAGCAGCGTGCAAATA-3′, 5′-GCATTGGGGCTGGTGTTATGA-3′, 58 °C; *C9orf72*: 5′-TGTGACAGTTGGAATGCAGTGA-3′, 5′-GCCACTTAAAGCAATCTCTGTCTTG-3′, 59 °C; *ACTB*: 5′-GTTACAGGAAGTCCCTTGCCATCC-3′, 5′-CACCTCCCCTGTGTGGACTTGGG-3′, 59 °C.

### RNA sequencing

Total RNA from iPSC-derived MNs was assessed for quality (Agilent Bionalyzer) and quantity (Invitrogen Qubit) before library preparation. Illumina libraries were prepared from 1 µg of total RNA using TruSeq RNA Sample Prep Kit v2 with a 10 cycle enrichment step as per the manufacturer’s recommendations. Final libraries were pooled in equimolar proportions before Illumina sequencing on a HiSeq 2500 platform using 75 base paired-end reads.

Reads were mapped to the primary assembly of the human (hg38) reference genome contained in Ensembl release 83. Alignment was performed with STAR, version 2.4.0i^70^. Tables of per-gene read counts were generated from the mapped reads with featureCounts version 1.4.6-p2^71^. Differential expression analysis was then performed using DESeq2^72^ (R package version 1.10.0).

### Immunocytochemistry

Cells were cultured depending on the experiment, washed once with PBS to remove media components, and fixed with 4% PFA for 20 min at room temperature. Cells were washed three times with PBS and blocking buffer containing 3% goat serum and 0.25% Triton X-100 in TBST (TBS-Tween) subsequently added for 30 min. Primary antibodies diluted in blocking buffer were then added and incubated overnight at 4 °C. Cells were then washed three times with TBS-Tween, and corresponding secondary antibodies coupled with fluorophores were added and incubated at room temperature for 1 h. Coverslips were washed and mounted on glass slides with FluorSave^TM^ reagent and imaged using Zeiss observer Z1. Images for cell count and neurite outgrowth were analyzed using ImageJ. Antibodies used in this study are as follows: MAP2 1:1000 (Sigma M9942), SMI312 1:1000 (Abcam ab24574), Tau 1:2000 (DAKO A0024), ISL1/2 1:50 (DSHB 39.4D5), ChAT 1:250 (EMDMillipore AB144P).

### Western blot analysis

MNs were cultured for 3 weeks and lysed directly with 2 × Laemmli buffer (125 mM Tris HCl pH 6.8, 4% SDS, 10% β−mercaptoethanol, 20% glycerol, and 0.004% bromophenol blue) and boiled for 10 min at 99 °C. Proteins were then run on a SDS-Poly acrylamide gel electrophoresis, blotted onto a nitrocellulose membrane. Membranes were then probed with respective primary and secondary antibodies and developed using ECL or ECL Advance (GE Healthcare). Blots were scanned and densitometry analysis was performed using ImageJ. Antibodies used GAPDH (1:5000), GluA1 (1:1000), C9ORF72 (1:2000).

### RNA fluorescence in situ hybridization

FISH analysis was performed using an Alexa546-conjugated (GGCCCC)_4_ oligoneucleotide probe (IDT). Briefly, cells on glass coverslips were fixed in 4% paraformaldehyde for 15 min, permeabilized in 70% ethanol at 4 °C overnight, incubated with 50% formamide diluted with 2X SSC for 10 min at room temperature and hybridized for 2 h at 45 °C with the probe (0.16 ng/µl) in hybridization buffer consisting of 50% formamide, 10% dextran sulfate, yeast transfer RNA (1 mg/ml), salmon sperm DNA (1 mg/ml). The cells were washed twice with 50% formamide diluted with 2 × SSC for 30 min at 37 °C and once with 2X SSC at room temperature for 30 min. Immunocytochemistry was performed as described above.

### Electrophysiology

Experiments investigating excitability were performed as previously described^26^. In short, whole-cell patch-clamp recordings were made in the current-clamp mode to determine firing properties of MNs. Recordings were performed using electrodes filled with internal pipette solution contained (in mM): 140 potassium methane-sulfonate, 10 NaCl, 1 CaCl_2_, 10 HEPES, 1 EGTA, 3 Mg_2_-ATP, and 0.4 Na_3_-GTP, pH 7.2–7.3 (adjusted with KOH; osmolarity adjusted to 300 mOsm with sucrose). Cells were bathed in an extracellular recording comprising (in mM): 127 NaCl, 3 KCl, 2 CaCl_2_, 1 MgCl_2_, 26 NaHCO_3_, 1.25 NaH_2_PO_4_, and 10 D-glucose (equilibrated with 95% O_2_ and 5% CO_2_, pH 7.45; osmolarity, 310 mOsm). Recordings were low-pass filtered at 4 kHz and digitized at 50 kHz using pClamp software (Axon Instruments). F–I relationships were generated by recording the responses to a series of 1 s duration current steps of 5–65 pA (10 pA increments).

For other electrophysiological experiments, recordings were performed as decribed^33^ using electrodes filled with (in mM): 155 K-gluconate, 2 MgCl_2_, 10 Na-HEPES, 10 Na-PiCreatine, 2 Mg_2_-ATP, and 0.3 Na_3_-GTP, pH 7.3, 300 mOsm. Cells were bathed in an extracellular recording comprising (in mM): 152 NaCl, 2.8 KCl, 10 HEPES, 2 CaCl_2_, 10 glucose, pH 7.3, 320–330 mOsm. Current and voltage measurements were typically low-pass filtered online at 2 kHz, digitized at 10 kHz and recorded to computer using the WinEDR V2 7.6 Electrophysiology Data Recorder (J. Dempster, Department of Physiology and Pharmacology, University of Strathclyde, UK; www.strath.ac.uk/Departments/PhysPharm/).

Non-stationary fluctuation analysis of slowly rising whole-cell currents evoked by AMPA were used to estimate the AMPAR unitary single-channel current. The analysis of AC and DC currents was performed as described previously in detail^73^ with the exception that the AC-coupled signal was filtered with a 1–1200 Hz band-pass frequency.

All experiments were carried out at room temperature. Patch electrodes were manufactured from borosilicate glass to have resistances of 3–6 MΩ. Recordings were made with a series resistance less than 25 MΩ. Reported potential values are corrected for liquid junction potential.

### Excitotoxicity assay

MNs (25,000 per well of a 96-well plate) were cultured for 1 or 3 week/s. Before the assay was performed, cells were further cultured overnight with minimal essential media containing neurobasal, B27, and N2. Live cells were labeled with 4 µM Calcein-AM (Invitrogen) for 5 min and imaged using Zeiss Observer Z1. Cells were then treated in the presence of cyclothiazide (100 µM) with either AMPA (100 µM) or AMPA/CNQX (30 µM) or AMPA/JTX (1 µM) for 24 h. Following treatment, cells were labeled again with Calcein AM and reimaged. Cell counts before and after treatment were analyzed using ImageJ.

### Human post-mortem tissue analysis

Formalin-fixed paraffin-embedded cervical spinal cord and prefrontal cortex (BA9) from three ALS patients carrying the *C9ORF72* repeat expansion and three age and sex-matched controls were sectioned at 5-μm thickness on to superfrost slides. BaseScope reagents (Advanced Cell Diagnostics) were used as per manufacturer’s guidelines according to the original protocol^74^. In brief, following deparafinisation, tissue was incubated with hydrogen peroxide for 10 min at room temperature and target antigen retrieval was performed by submerging slides in BaseScope 1 × target retrieval reagent at 99 °C in a Braun Multiquick FS 20 steamer for 15 min. The tissue was then permeabilised using BaseScope protease III at 40 °C for 30 min. Probe hybridization was then performed by incubating the slides with four drops of custom-designed BaseScope probe for AMPAR subunits, negative control probe (DapB) or positive control (PPIB) probe for 2 h at 40 °C. Following successive probe amplification steps, transcripts were detected using the BaseScope RED detection kit and slides were counterstained using 50% Gills haematoxylin and 0.02% ammonium water. The slides were then cleared in xylene and mounted with a 24 × 50 mm coverslip using two drops of VectaMount mounting medium. Sections were then imaged at 80× magnification on a NanoZoomer and relative number of transcripts was quantified by counting the dots observed in the cell manually by two independent neuropathologist. Three random fields were chosen and ten cells from each field were counted (in total 30 cells per post-mortem sample) and averaged for each patient. Data were plotted as mean number of transcripts (dots per cell) per post-mortem sample.

### Data analysis

Statistical analysis was performed using GraphPad Prism software. Data are represented as mean ± s.e.m. *p < 0.05, **p < 0.01, ***p < 0.001, ****p < 0.0001, one-way ANOVA with Bonferroni’s multiple comparisons test or uncorrected Fisher’s LSD test.

### Data availability

The data that support the findings of this study are available from the corresponding author upon reasonable request.

## Electronic supplementary material

Supplementary Information

Description of Additional Supplementary Files

Supplementary Data 1

Supplementary Data 2

## References

[1] DeJesus-Hernandez M (2011). Expanded GGGGCC hexanucleotide repeat in noncoding region of C9ORF72 causes chromosome 9p-linked FTD and ALS. Neuron.

[2] Smith BN (2013). The C9ORF72 expansion mutation is a common cause of ALS+/-FTD in Europe and has a single founder. Eur. J. Hum. Genet..

[3] Rothstein, J. D. Excitotoxic mechanisms in the pathogenesis of amyotrophic lateral sclerosis. *Adv. Neurol.* **68**, 7–20, discussion 21–27 (1995). 8787245

[4] Rothstein JD, Martin LJ, Kuncl RW (1992). Decreased glutamate transport by the brain and spinal cord in amyotrophic lateral sclerosis. N. Engl. J. Med..

[5] Rothstein JD (1990). Abnormal excitatory amino acid metabolism in amyotrophic lateral sclerosis. Ann. Neurol..

[6] Couratier P, Hugon J, Sindou P, Vallat JM, Dumas M (1993). Cell culture evidence for neuronal degeneration in amyotrophic lateral sclerosis being linked to glutamate AMPA/kainate receptors. Lancet.

[7] Day NC (1995). Distribution of AMPA-selective glutamate receptor subunits in the human hippocampus and cerebellum. Brain Res. Mol. Brain Res..

[8] Tortarolo M (2006). Glutamate AMPA receptors change in motor neurons of SOD1G93A transgenic mice and their inhibition by a noncompetitive antagonist ameliorates the progression of amytrophic lateral sclerosis-like disease. J. Neurosci. Res..

[9] Petri S (2005). The cellular mRNA expression of GABA and glutamate receptors in spinal motor neurons of SOD1 mice. J. Neurol. Sci..

[10] Rembach A (2004). Antisense peptide nucleic acid targeting GluR3 delays disease onset and progression in the SOD1 G93A mouse model of familial ALS. J. Neurosci. Res..

[11] Zhao P, Ignacio S, Beattie EC, Abood ME (2008). Altered presymptomatic AMPA and cannabinoid receptor trafficking in motor neurons of ALS model mice: implications for excitotoxicity. Eur. J. Neurosci..

[12] Van Den Bosch L, Vandenberghe W, Klaassen H, Van Houtte E, Robberecht W (2000). Ca(2+)-permeable AMPA receptors and selective vulnerability of motor neurons. J. Neurol. Sci..

[13] Kuner R (2005). Late-onset motoneuron disease caused by a functionally modified AMPA receptor subunit. Proc. Natl Acad. Sci. USA.

[14] Vandenberghe W, Ihle EC, Patneau DK, Robberecht W, Brorson JR (2000). AMPA receptor current density, not desensitization, predicts selective motoneuron vulnerability. J. Neurosci..

[15] Kawahara Y (2004). Glutamate receptors: RNA editing and death of motor neurons. Nature.

[16] Kawahara Y (2004). Regulation of glutamate receptor RNA editing and ADAR mRNA expression in developing human normal and Down’s syndrome brains. Brain Res. Dev. Brain Res..

[17] Sareen D (2013). Targeting RNA foci in iPSC-derived motor neurons from ALS patients with a C9ORF72 repeat expansion. Sci. Transl. Med..

[18] Donnelly CJ (2013). RNA toxicity from the ALS/FTD C9ORF72 expansion is mitigated by antisense intervention. Neuron.

[19] Sandoe J, Eggan K (2013). Opportunities and challenges of pluripotent stem cell neurodegenerative disease models. Nat. Neurosci..

[20] Park CY (2015). Reversion of FMR1 methylation and silencing by editing the triplet repeats in fragile X iPSC-derived neurons. Cell Rep..

[21] Reinhardt P (2013). Genetic correction of a LRRK2 mutation in human iPSCs links parkinsonian neurodegeneration to ERK-dependent changes in gene expression. Cell Stem Cell.

[22] Firth AL (2015). Functional gene correction for cystic fibrosis in lung epithelial cells generated from patient iPSCs. Cell Rep..

[23] Pattanayak V (2013). High-throughput profiling of off-target DNA cleavage reveals RNA-programmed Cas9 nuclease specificity. Nat. Biotechnol..

[24] Maury Y (2015). Combinatorial analysis of developmental cues efficiently converts human pluripotent stem cells into multiple neuronal subtypes. Nat. Biotechnol..

[25] Haeusler AR, Donnelly CJ, Rothstein JD (2016). The expanding biology of the C9orf72 nucleotide repeat expansion in neurodegenerative disease. Nat. Rev. Neurosci..

[26] Devlin AC (2015). Human iPSC-derived motoneurons harbouring TARDBP or C9ORF72 ALS mutations are dysfunctional despite maintaining viability. Nat. Commun..

[27] Wainger BJ (2014). Intrinsic membrane hyperexcitability of amyotrophic lateral sclerosis patient-derived motor neurons. Cell Rep..

[28] Foran E, Trotti D (2009). Glutamate transporters and the excitotoxic path to motor neuron degeneration in amyotrophic lateral sclerosis. Antioxid. Redox Signal..

[29] Traynelis SF (2010). Glutamate receptor ion channels: structure, regulation, and function. Pharmacol. Rev..

[30] Sommer B, Kohler M, Sprengel R, Seeburg PH (1991). RNA editing in brain controls a determinant of ion flow in glutamate-gated channels. Cell.

[31] Iino M, Koike M, Isa T, Ozawa S (1996). Voltage-dependent blockage of Ca(2+)-permeable AMPA receptors by joro spider toxin in cultured rat hippocampal neurones. J. Physiol..

[32] Bilican B (2014). Physiological normoxia and absence of EGF is required for the long-term propagation of anterior neural precursors from human pluripotent cells. PLoS ONE.

[33] Livesey MR (2014). Maturation of AMPAR composition and the GABAAR reversal potential in hPSC-derived cortical neurons. J. Neurosci..

[34] Rekling JC, Funk GD, Bayliss DA, Dong XW, Feldman JL (2000). Synaptic control of motoneuronal excitability. Physiol. Rev..

[35] Williams TL, Ince PG, Oakley AE, Shaw PJ (1996). An immunocytochemical study of the distribution of AMPA selective glutamate receptor subunits in the normal human motor system. Neuroscience.

[36] Tran H (2015). Differential toxicity of nuclear RNA foci versus dipeptide repeat proteins in a drosophila model of C9ORF72 FTD/ALS. Neuron.

[37] Prudencio M (2015). Distinct brain transcriptome profiles in C9orf72-associated and sporadic ALS. Nat. Neurosci..

[38] Zhang K (2015). The C9orf72 repeat expansion disrupts nucleocytoplasmic transport. Nature.

[39] Ho R (2016). ALS disrupts spinal motor neuron maturation and aging pathways within gene co-expression networks. Nat. Neurosci..

[40] Mizielinska S (2014). C9orf72 repeat expansions cause neurodegeneration in Drosophila through arginine-rich proteins. Science.

[41] Sances S (2016). Modeling ALS with motor neurons derived from human induced pluripotent stem cells. Nat. Neurosci..

[42] Fritz E (2013). Mutant SOD1-expressing astrocytes release toxic factors that trigger motoneuron death by inducing hyperexcitability. J. Neurophysiol..

[43] Heath PR, Tomkins J, Ince PG, Shaw PJ (2002). Quantitative assessment of AMPA receptor mRNA in human spinal motor neurons isolated by laser capture microdissection. Neuroreport.

[44] Hideyama T (2010). Induced loss of ADAR2 engenders slow death of motor neurons from Q/R site-unedited GluR2. J. Neurosci..

[45] Kawahara Y (2003). Human spinal motoneurons express low relative abundance of GluR2 mRNA: an implication for excitotoxicity in ALS. J. Neurochem..

[46] Kawahara Y (2006). Underediting of GluR2 mRNA, a neuronal death inducing molecular change in sporadic ALS, does not occur in motor neurons in ALS1 or SBMA. Neurosci. Res..

[47] Takuma H, Kwak S, Yoshizawa T, Kanazawa I (1999). Reduction of GluR2 RNA editing, a molecular change that increases calcium influx through AMPA receptors, selective in the spinal ventral gray of patients with amyotrophic lateral sclerosis. Ann. Neurol..

[48] Pieri M (2003). Altered excitability of motor neurons in a transgenic mouse model of familial amyotrophic lateral sclerosis. Neurosci. Lett..

[49] Spalloni A (2006). Molecular and synaptic changes in the hippocampus underlying superior spatial abilities in pre-symptomatic G93A +/+ mice overexpressing the human Cu/Zn superoxide dismutase (Gly93 > ALA) mutation. Exp. Neurol..

[50] Gascon E (2014). Alterations in microRNA-124 and AMPA receptors contribute to social behavioral deficits in frontotemporal dementia. Nat. Med..

[51] Livesey MR (2016). Maturation and electrophysiological properties of human pluripotent stem cell-derived oligodendrocytes. Stem Cells.

[52] Cleveland DW, Rothstein JD (2001). From Charcot to Lou Gehrig: deciphering selective motor neuron death in ALS. Nat. Rev. Neurosci..

[53] Van Den Bosch L, Van Damme P (2006). The role of excitotoxicity in the pathogenesis of amyotrophic lateral sclerosis. Biochim. Biophys. Acta.

[54] Li YH, Zhang N, Wang YN, Shen Y, Wang Y (2016). Multiple faces of protein interacting with C kinase 1 (PICK1): structure, function, and diseases. Neurochem. Int..

[55] Cristofani R (2018). The small heat shock protein B8 (HSPB8) efficiently removes aggregating species of dipeptides produced in C9ORF72-related neurodegenerative diseases. Cell Stress Chaperones.

[56] Crippa V (2016). The chaperone HSPB8 reduces the accumulation of truncated TDP-43 species in cells and protects against TDP-43-mediated toxicity. Hum. Mol. Genet..

[57] Crippa V (2010). The small heat shock protein B8 (HspB8) promotes autophagic removal of misfolded proteins involved in amyotrophic lateral sclerosis (ALS). Hum. Mol. Genet..

[58] Lee AH, Iwakoshi NN, Glimcher LH (2003). XBP-1 regulates a subset of endoplasmic reticulum resident chaperone genes in the unfolded protein response. Mol. Cell. Biol..

[59] Nyathi Y, Wilkinson BM, Pool MR (2013). Co-translational targeting and translocation of proteins to the endoplasmic reticulum. Biochim. Biophys. Acta.

[60] Hori O (2006). Deletion of SERP1/RAMP4, a component of the endoplasmic reticulum (ER) translocation sites, leads to ER stress. Mol. Cell. Biol..

[61] Yamaguchi A (1999). Stress-associated endoplasmic reticulum protein 1 (SERP1)/ribosome-associated membrane protein 4 (RAMP4) stabilizes membrane proteins during stress and facilitates subsequent glycosylation. J. Cell Biol..

[62] McCaffrey K, Braakman I (2016). Protein quality control at the endoplasmic reticulum. Essays Biochem..

[63] Dafinca R (2016). C9orf72 hexanucleotide expansions are associated with altered endoplasmic reticulum calcium homeostasis and stress granule formation in induced pluripotent stem cell-derived neurons from patients with amyotrophic lateral sclerosis and frontotemporal dementia. Stem Cells.

[64] Haeusler AR (2014). C9orf72 nucleotide repeat structures initiate molecular cascades of disease. Nature.

[65] Boeynaems S, Bogaert E, Van Damme P, Van Den Bosch L (2016). Inside out: the role of nucleocytoplasmic transport in ALS and FTLD. Acta Neuropathol..

[66] Bahar E, Kim H, Yoon H (2016). ER stress-mediated signaling: action potential and Ca(2+) as key players. Int. J. Mol. Sci..

[67] Vandenberghe W, Nicoll RA, Bredt DS (2005). Interaction with the unfolded protein response reveals a role for stargazin in biosynthetic AMPA receptor transport. J. Neurosci..

[68] Ran FA (2013). Genome engineering using the CRISPR-Cas9 system. Nat. Protoc..

[69] Almeida S (2013). Modeling key pathological features of frontotemporal dementia with C9ORF72 repeat expansion in iPSC-derived human neurons. Acta Neuropathol..

[70] Dobin A (2013). STAR: ultrafast universal RNA-seq aligner. Bioinformatics.

[71] Liao Y, Smyth GK, Shi W (2014). featureCounts: an efficient general purpose program for assigning sequence reads to genomic features. Bioinformatics.

[72] Love MI, Huber W, Anders S (2014). Moderated estimation of fold change and dispersion for RNA-seq data with DESeq2. Genome Biol..

[73] Brown AM, Hope AG, Lambert JJ, Peters JA (1998). Ion permeation and conduction in a human recombinant 5-HT3 receptor subunit (h5-HT3A). J. Physiol..

[74] Wang F (2012). RNAscope: a novel in situ RNA analysis platform for formalin-fixed, paraffin-embedded tissues. J. Mol. Diagn..

