# Supplementary material for: C9ORF72 repeat expansion causes vulnerability of motor neurons to Ca2+-permeable AMPA receptor-mediated excitotoxicity
Source: Nat Commun. 2018 Jan 24;9:347. doi: 10.1038/s41467-017-02729-0 (PMC5783946; doi:10.1038/s41467-017-02729-0)
Supplement: Supplementary file 1 — Supplementary Information [file 41467_2017_2729_MOESM1_ESM.pdf]

Supplementary Figure 1

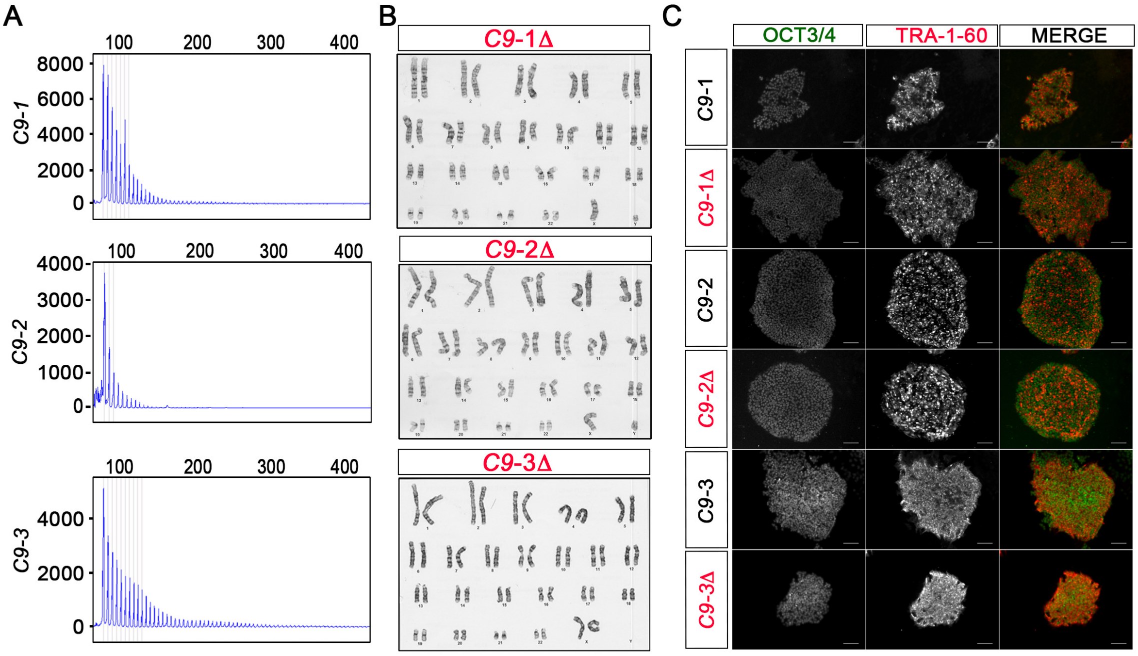

D

<u>C9-gRNA-1 sequence : AACTCAGGAGTCGCGCGCTA</u>

C9_Fw_chr2:-231921408 : AGGTCAGGAGTCCCGCGCAA

```
Control  AAACCTCCTCGAGGTCAGGAGTCCCGCGCAACGGAACACTGGTCT
C9-1Δ    AAACCTCCTCGAGGTCAGGAGTCCCGCGCAACGGAACACTGGTCT
C9-2Δ    AAACCTCCTCGAGGTCAGGAGTCCCGCGCAACGGAACACTGGTCT
C9-3Δ    AAACCTCCTCGAGGTCAGGAGTCCCGCGCAACGGAACACTGGTCT
         *********************************************
```

C9_Fw_chr7:-151551819 : ATCACAGGAGGCGCGCCCTA

```
Control  GTTGTGTGAGAATCACAGGAGGCGCGCCCTAAGGACCAGGCACCC
C9-1Δ    GTTGTGTGAGAATCACAGGAGGCGCGCCCTAAGGACCAGGCACCC
C9-2Δ    GTTGTGTGAGAATCACAGGAGGCGCGCCCTAAGGACCAGGCACCC
C9-3Δ    GTTGTGTGAGAATCACAGGAGGCGCGCCCTAAGGACCAGGCACCC
         *********************************************
```

<u>C9-gRNA-2 sequence : GGCCCGCCCCGACCACGCCC</u>

C9_Rev_chr3:+98241587_NM_001040199: CGCCCAGCCCGACCACGCCC

```
Control  CGCTCGGAACCCGCCCAGCCCGACCACGCCCGGGAAGGGGGCCCC
C9-1Δ    CGCTCGGAACCCGCCCAGCCCGACCACGCCCGGGAAGGGGGCCCC
C9-2Δ    CGCTCGGAACCCGCCCAGCCCGACCACGCCCGGGAAGGGGGCCCC
C9-3Δ    CGCTCGGAACCCGCCCAGCCCGACCACGCCCGGGAAGGGGGCCCC
         *********************************************
```

C9_Rev_chr22:-37731099 : GCCCCGCTCCGCCCACGCCC

```
Control  TTCTTCCTGGATGCCCCGCTCCGCCCACGCCCAAGTTCTCCGGCA
C9-1Δ    TTCTTCCTGGATGCCCCGCTCCGCCCACGCCCAAGTTCTCCGGCA
C9-2Δ    TTCTTCCTGGATGCCCCGCTCCGCCCACGCCCAAGTTCTCCGGCA
C9-3Δ    TTCTTCCTGGATGCCCCGCTCCGCCCACGCCCAAGTTCTCCGGCA
         *********************************************
```

SUPPLEMENTARY FIGURE 1. A, Repeat primed PCR data depicting the extent of G_4C_2 repeat expansions on mutant *C9ORF72* iPSCs. **B,** Karyotype of C9-1Δ, C9-2Δ and C9-3Δ iPSC lines. **C,** C9-1Δ, C9-2Δ and C9-3Δ iPSCs expresses various pluripotency markers (Oct3/4, Tra-1-60). **D**, Sanger sequencing to analyse off-target effects of gRNAs in C9-1Δ, C9-2Δ and C9-3Δ iPSCs.

Supplementary Figure 2

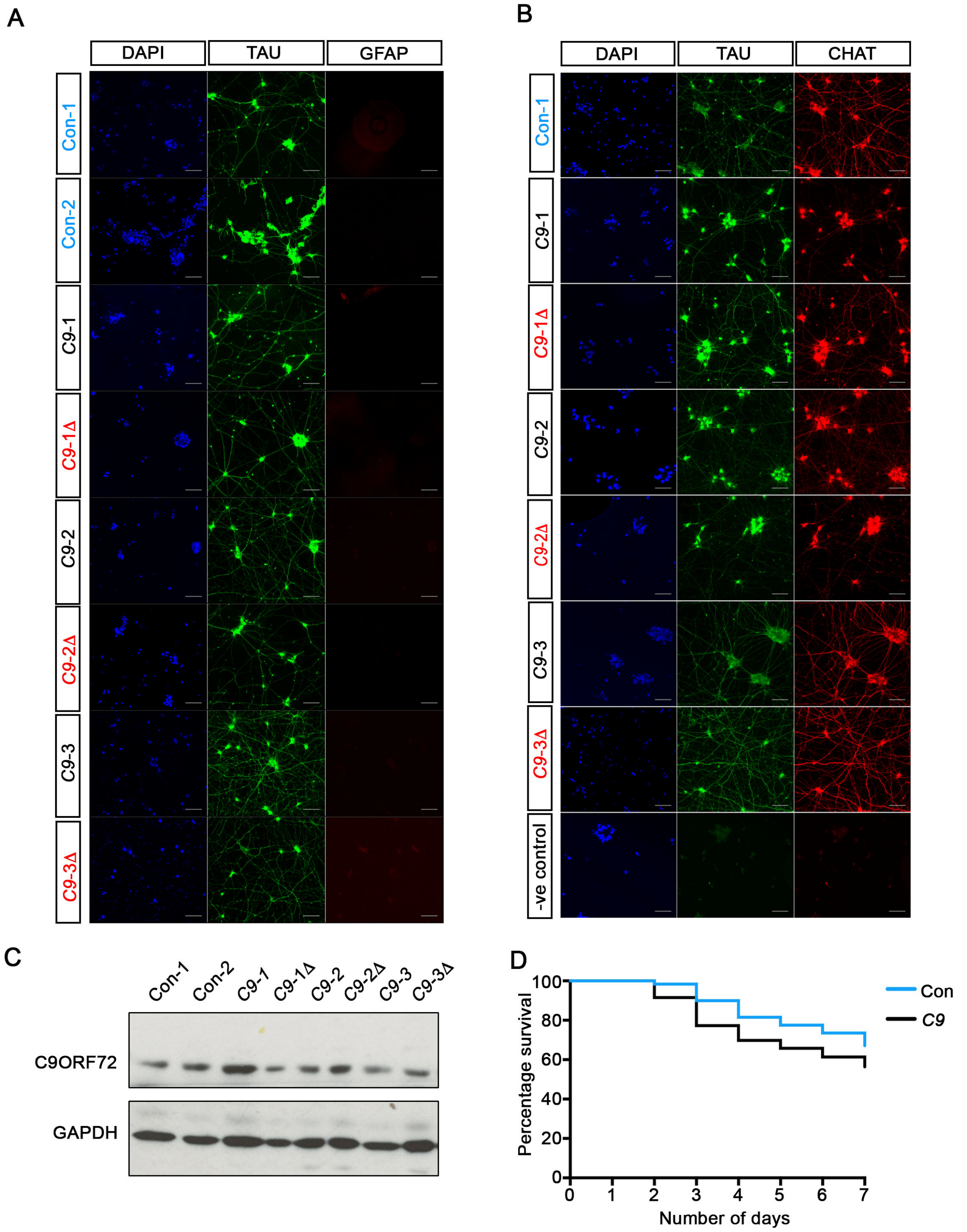

SUPPLEMENTARY FIGURE 2. A, Representative immunofluorescence images of MNs labelled with TAU (neuronal marker) and GFAP (astrocyte marker), scale bar: 50 µm). **B,** Representative image of CHAT immunostaining in 3 week old MNs, scale bar: 50 µm. **C,** Representative Western blot analysis of C9ORF72 protein level in control, mutant *C9ORF72* and C9-Δ iPSC derived MNs. **D**, Longitudinal survival analysis of MNs derived from control and mutant *C9ORF72* MNs showed that the G_4C_2 expansion repeats did not affect survival of MNs. Con-1, 53.6±8.5% N=3; Con-2, 63.9±8.9% N=3; C9-1, 53.6±4.9% N=3; C9-3, 46.6±10.4% N=3 at least 35 cells per experiment

Supplementary Figure 3

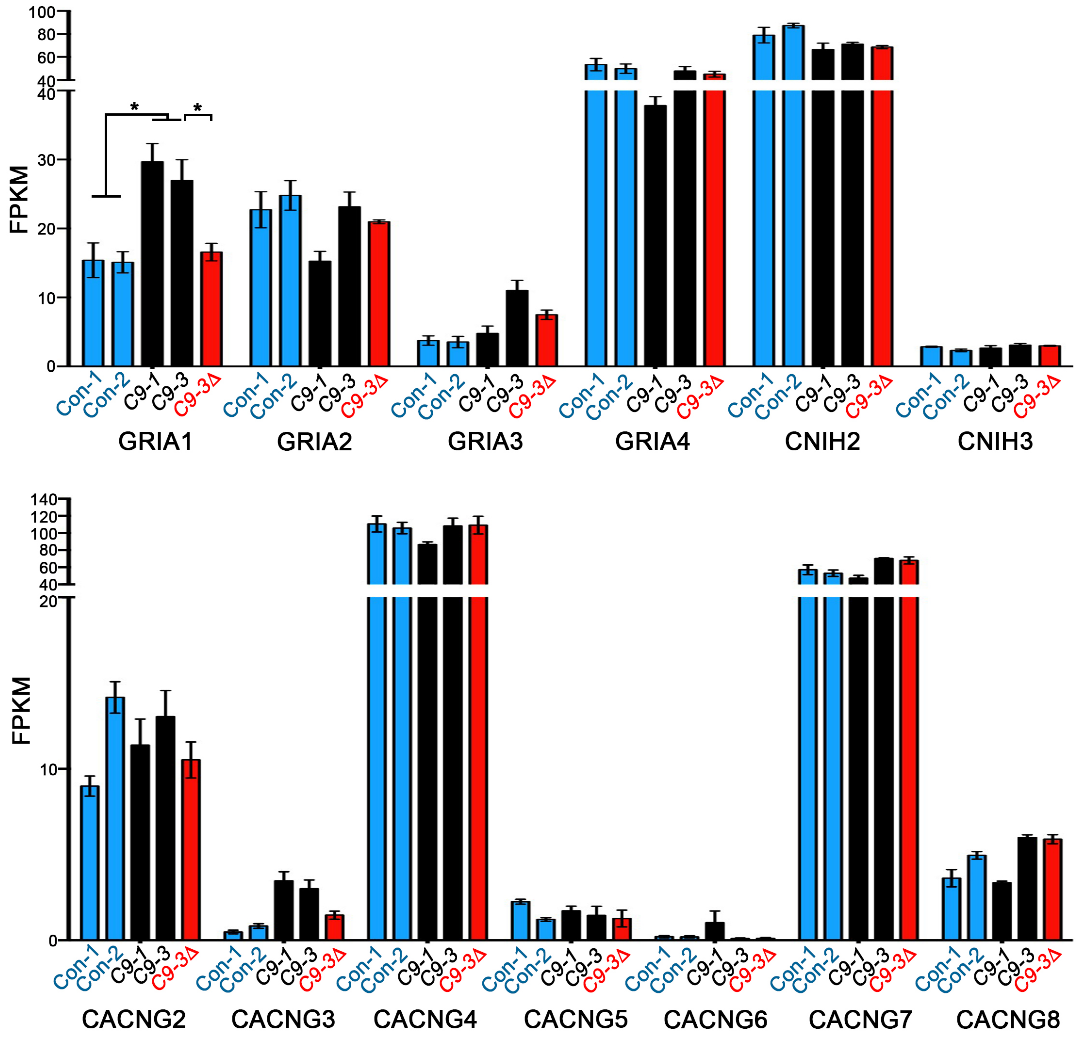

SUPPLEMENTARY FIGURE 3. RNA-Seq analysis of AMPAR subunits and auxiliary subunits performed on 3-week old control, mutant *C9ORF72*, C9-3Δ MNs (N=3 for all genotypes).

Supplementary Figure 4

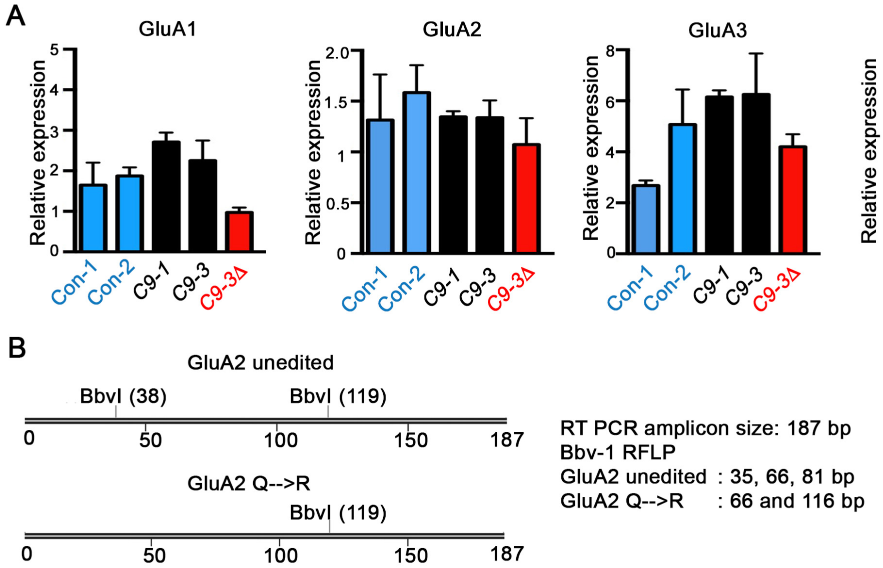

SUPPLEMENTARY FIGURE 4. A, Relative mRNA expression of AMPAR subunits in all cultures examined at Week 1 after differentiation. Data represented as mean ± s.e.m, Con-1, N=3;Con-2, N=5; C9-1, N=3; C9-3, N=4; C9-3Δ, N=4. **B,** Restriction fragment length polymorphism (RFLP) strategy to detect the GluA2 A→I mRNA post-transcriptional editing using Bbv1 endonuclease.

Supplementary Figure 5

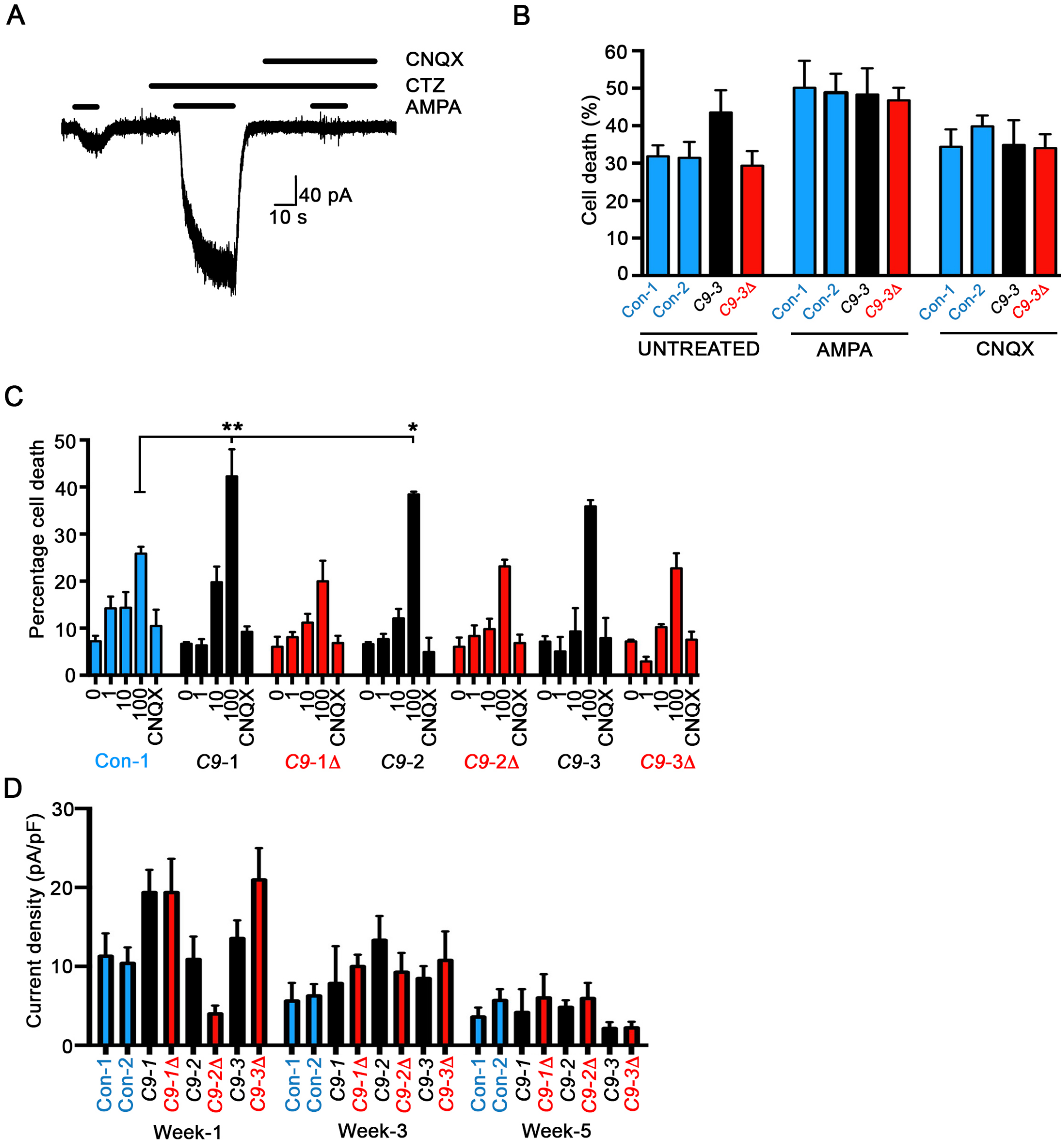

SUPPLEMENTARY FIGURE 5. A, Whole-cell voltage-clamp recording conducted at -74 mV showing the potentiation of AMPA (10 µM)-mediated currents by cyclothiazide (10 µM), an AMPAR-selective allosteric potentiator, and subsequent block by CNQX (10 µM). **B,** Mean percentage cytotoxicity of 1 week old MNs treated with AMPA and AMPA+CNQX for 24 h. Data represented as mean ± s.e.m; Con-1, N=3;Con-2, N=3; C9-3, N=3; C9-3Δ, N=3. **C,** Mean percentage cell death of week 3 MNs treated with different concentrations of AMPA (in the presence of CTZ) for 24 h. Data represented as mean ± s.e.m; Con-1, N=3;Con-2, N=3; C9-1, N=3; C9-1Δ, N=3; C9-2, N=3; C9-2Δ, N=3; C9-3, N=3; C9-3Δ, N=3. Statistical comparisons; one-way ANOVA with uncorrected Fisher's LSD. **D**, Mean AMPAR current density measurements in all lines examined at Week 1, 3 and 5 after differentiation. For all lines at each time point n=3-16, *N*=1-4. Statistical difference was not found between patient and healthy/isogenic MNs (one-way ANOVA with Bonferroni's *post hoc* test)

Supplementary Figure 6

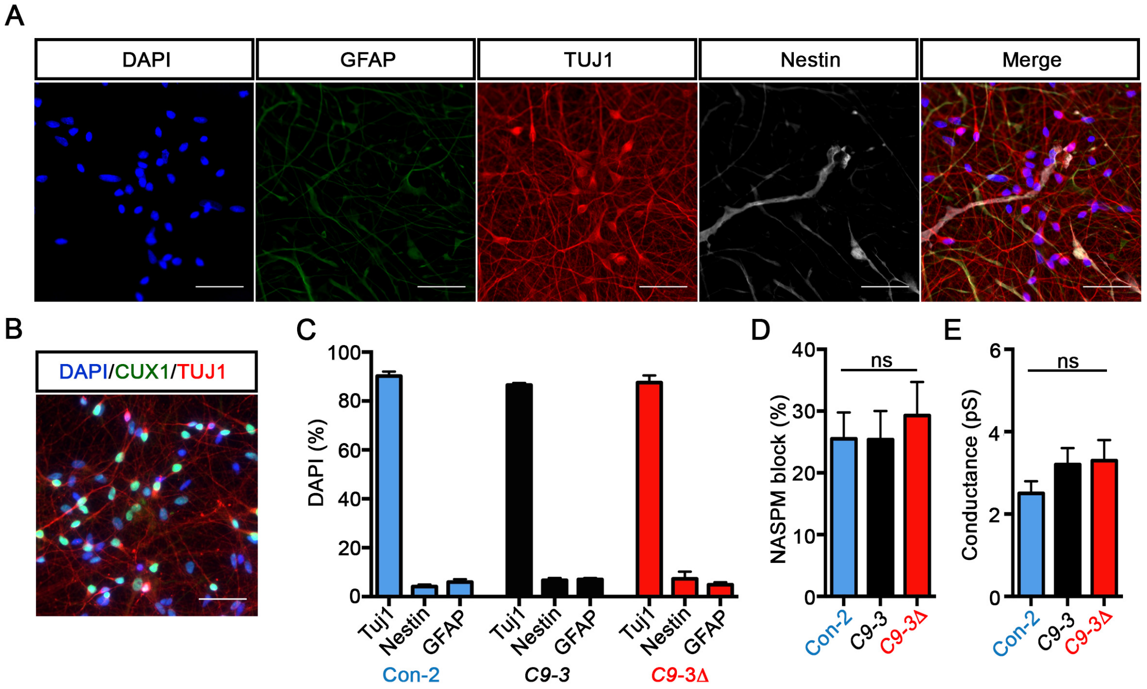

SUPPLEMENTARY FIGURE 6. A-B, Representative images of stainings for 5-week old iPSC derived cortical neurons with Tuj1/GFAP/Nestin (A) and CUX1/Tuj1 (B). **C**, Basic % Tuj1/Nestin/GFAP with respect to DAPI at 5 weeks. **D**, Mean percentage NASPM block of AMPA-induced currents in all cortical neurons examined at Week 5 after differentiation. No significant difference was observed between lines (data represented as as mean ± s.e.m; Con-2, n=9, N=3; C9-3, n=7, N=3; C9-3Δ, n=8, N=3). **E**, Mean estimated AMPAR γ in all lines examined at Week 5 after differentiation. No significant difference was observed between lines (data represented as mean ± s.e.m; Con-2, n=14, N=3; C9-3, n=17, N=3; C9-3Δ, n=17, N=3).

Supplementary Figure 7

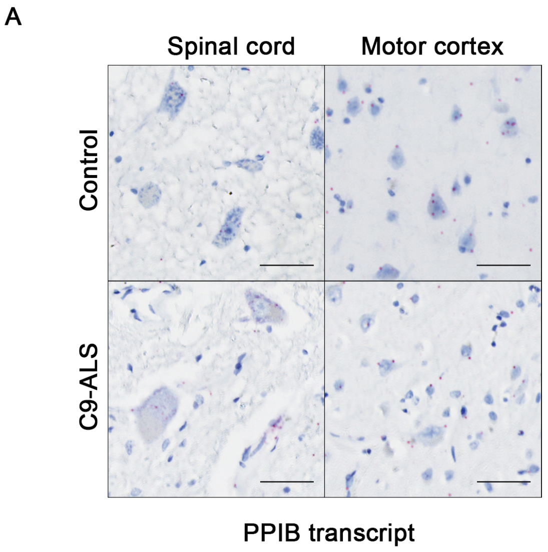

SUPPLEMENTARY FIGURE 7. Positive control staining of PPIB in human post mortem sections. Representative RNAScope images showing the expression of PPIB in the spinal cord and prefrontal cortex of the same patients presented in Figure 7. Scale bar, 50 µm.

Supplementary Figure 8

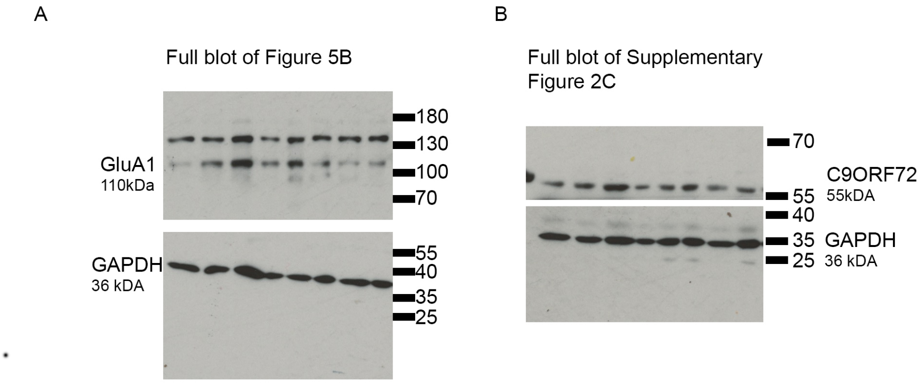

SUPPLEMENTARY FIGURE 8. Full blot of Figure 5B and Supplementary Figure 2C

SUPPLEMENTARY TABLE 1: Intrinsic properties of iPSC-derived motor neurons

iPSC-derived line	Week	Input Resistance (MΩ)	Resting Membrane Potential (mV)	Whole-Cell Capacitance (pF)	Frequency-Input slopes
Con-1	1<br>3<br>5	1982 ± 172<br>1206 ± 169[a]<br>701 ± 66[a,b]	-56.6 ± 2.1<br>-55.8 ± 2.4<br>-60.5 ± 1.6	16.7 ± 1.6<br>18.5 ± 2.0<br>23.0 ± 1.9	0.121 ± 0.07
Con-2	1<br>3<br>5	1456 ± 156<br>841 ± 88[a]<br>693 ± 50[a]	-49.2 ± 2.5<br>-51.6 ± 2.0<br>-59.1 ± 21.6	15.3 ± 0.8<br>20.3 ± 1.7<br>28.1 ± 2.1[a]	0.175 ± 0.03
C9-1	1<br>3<br>5	1596 ± 189<br>1393 ± 139<br>1003 ± 85[a]	-53.7 ± 1.9<br>-59.6 ± 1.9<br>-59.9 ± 1.4	14.8 ± 1.3<br>23.7 ± 2.4<br>25.9 ± 2.0[a]	0.156 ± 0.03
C9-1Δ	1<br>3<br>5	1159 ± 96<br>983 ± 144<br>793 ± 59[a]	-39.3 ± 1.2<br>-53.8 ± 1.9[a]<br>-52.8 ± 1.4[a]	11.8 ± 1.1<br>24.4 ± 4.5[a]<br>18.1 ± 1.4	0.163 ± 0.06
C9-2	1<br>3<br>5	1824 ± 195<br>801 ± 107<br>682 ± 43[b]	-37.8 ± 2.1<br>-46.2 ± 3.8<br>-51.7 ± 2.4[a,b]	19.4 ± 2.4<br>34.7 ± 3.4[a]<br>40.7 ± 2.0[a]	0.136 ± 0.04
C9-2Δ	1<br>3<br>5	1406 ± 183<br>685 ± 97[a]<br>646 ± 52[a]	-38.3 ± 2.1<br>-47.9 ± 2.6<br>-52.8 ± 1.4[a,b]	24.2 ± 2.3<br>29.4 ± 2.9<br>24.1 ± 2.7	0.082 ± 0.06
C9-3	1<br>3<br>5	1854 ± 218<br>1335 ± 140[a]<br>816 ± 54[a,b]	-54.9 ± 2.0<br>-52.2 ± 2.3<br>-59.1 ± 1.2[b]	17.0 ± 1.2<br>16.3 ± 1.5<br>23.1 ± 1.2[a,b]	0.130 ± 0.04
C9-3Δ	1<br>3<br>5	2118 ± 189<br>959 ± 108 [a]<br>757 ± 52[a]	-54.7 ± 2.1<br>-61.4 ± 2.7<br>-65.2 ± 1.1[a]	12.4 ± 1.0<br>27.6 ± 3.4[a]<br>26.9 ± 1.2[a]	0.221 ± 0.03

SUPPLEMENTARY TABLE 1. Intrinsic properties recorded from iPSC-derived motor neurons. Mean input resistance, resting membrane potential and whole-cell capacitance data for each line obtained at weeks 1, 3 and 5. Data points were obtained from a range of 6-99 cells derived from 2-9 *de novo* batches of cells. Statistical data (one-way ANOVA with Bonferroni's *post hoc* test) presented with respect week 1 ([a] signifies p>0.05) and week 3 data ([b] signifies p>0.05). Mean frequency-input slope data for repetitively firing Week 5 cells from each iPSC-derived line. Data obtained from 3-39 cells. Statistical significance is not observed between patient versus healthy/isogenic lines (one-way ANOVA with Bonferroni's *post hoc* test).

SUPPLEMENTARY DATA 1: List of genes that are significantly upregulated in *C9ORF72* mutant motor neurons (attached excel sheet)

SUPPLEMENTARY DATA 2: List of genes that are significantly downregulated in *C9ORF72* mutant motor neurons (attached excel sheet)
