# Supplementary material for: C9ORF72 repeat expansion causes vulnerability of motor neurons to Ca2+-permeable AMPA receptor-mediated excitotoxicity
Source: Nat Commun. 2018 Jan 24;9:347. doi: 10.1038/s41467-017-02729-0 (PMC5783946; doi:10.1038/s41467-017-02729-0)
Supplement: Supplementary file 2 — Description of Additional Supplementary Files [file 41467_2017_2729_MOESM2_ESM.pdf]

Description of Additional Supplementary Files

File Name: Supplementary Data 1
Description: List of genes that are significantly upregulated in C9ORF72 mutant motor neurons.

File Name: Supplementary Data 2
Description: List of genes that are significantly downregulated in C9ORF72 mutant motor neurons.
